# Archaeobotanical evidence supports indigenous cucurbit long-term use in the Mesoamerican Neotropics

**DOI:** 10.1038/s41598-024-60723-1

**Published:** 2024-05-13

**Authors:** Alejandra I. Domic, Amber M. VanDerwarker, Heather B. Thakar, Kenneth Hirth, José M. Capriles, Thomas K. Harper, Timothy E. Scheffler, Logan Kistler, Douglas J. Kennett

**Affiliations:** 1 Department of Anthropology, The Pennsylvania State University, University Park, PA 16802 USA; 2 Department of Geosciences, The Pennsylvania State University, University Park, PA 16802 USA; 3 Department of Anthropology, University of California, Santa Barbara, CA 93106 USA; 4 Department of Anthropology, Texas A&M University, College Station, TX 77843 USA; 5 tesARCH Services, Volcano, HI 96785 USA; 6 Department of Anthropology, Smithsonian National Museum of Natural History, Washington, DC USA

**Keywords:** Bottle gourd, Domestication, Mesoamerica, Squash, Agriculture, Archaeology, Plant evolution

## Abstract

The squash family (Cucurbitaceae) contains some of the most important crops cultivated worldwide and has played an important ecological, economic, and cultural role for millennia. In the American tropics, squashes were among the first cultivated crop species, but little is known about how their domestication unfolded. Here, we employ direct radiocarbon dating and morphological analyses of desiccated cucurbit seeds, rinds, and stems from El Gigante Rockshelter in Honduras to reconstruct human practices of selection and cultivation of *Lagenaria siceraria*, *Cucurbita pepo*, and *Cucurbita moschata*. Direct radiocarbon dating indicates that humans started using *Lagenaria* and wild *Cucurbita* starting ~ 10,950 calendar years before present (cal B.P.), primarily as watertight vessels and possibly as cooking and drinking containers. A rind directly dated to 11,150–10,765 cal B.P. represents the oldest known bottle gourd in the Americas. Domesticated *C. moschata* subsequently appeared ~ 4035 cal B.P., followed by domesticated *C. pepo* ~ 2190 cal B.P. associated with increasing evidence for their use as food crops. Multivariate statistical analysis of seed size and shape show that the archaeological *C. pepo* assemblage exhibits significant variability, representing at least three varieties: one similar to present-day zucchini, another like present-day vegetable marrow, and a native cultivar without modern analogs. Our archaeobotanical data supports the hypothesis that Indigenous cucurbit use started in the Early Holocene, and that agricultural complexity during the Late Holocene involved selective breeding that encouraged crop diversification.

## Introduction

The squash family (Cucurbitaceae) consists of nearly a thousand species worldwide, all of great ecological, economic and cultural importance^1^. At least six cucurbit species were domesticated in the Americas and are currently cultivated globally for their edible fruit and seeds^1^. *Lagenaria siceraria* (Molina) Standl. (bottle gourd), *Cucurbita pepo* L. (summer squash, acorn squash, jack-o-lantern pumpkins), *Cucurbita argyrosperma* K. Koch (cushaw), *Cucurbita moschata* Duchesne (butternut squash, commercial pie pumpkins), *Cucurbita ficifolia* Bouché (chilacayote, fig-leaf gourd), and *Cucurbita maxima* Duchesne (hubbard squashes, pumpkins). These cultivated cucurbits originated from different wild ancestors in different regions and constitute an important part of various cultural traditions across the Americas. Cultivated cucurbits are broadly characterized by large fruit sizes, variability in fruit shape and color, increased sugar and carotenoid content, decreased bitterness, and dominant upright growth^2^.

Archaeological evidence shows that cucurbits were among the first field crops cultivated in the Americas^3,4^. *L. siceraria* is native to Africa, but it arrived in the Americas during the Late Pleistocene and was likely domesticated independently in Eurasia, Africa, and the Americas^3–5^. Bottle gourds were widely used in the Americas during the Early Holocene and archaeological remains have been found in early archaeological contexts ranging from Florida (~ 10,000 cal B.P.), Mexico (~ 8000 cal B.P.), and Panama (~ 8300 cal B.P.) to Ecuador (9300 BP) and Peru (9000–5500 cal B.P.)^3,6–8^.

*Cucurbita pepo* was separately domesticated in at least two different regions, leading to distinct subspecies: *C. pepo* subsp*. ovifera* in eastern North America and *C. pepo* L. subsp*. pepo* in northeastern Mexico^9,10^. The initial domestication of *C. pepo* might have taken place near Guilá Naquitz and the Tehuacán valley (~ 10,000 cal B.P.) and subsequently diffused to central (7920 cal B.P.) and northern Mexico (6310 cal B.P.)^3,11–15^. *C. argyrosperma* was domesticated from *C. argyrosperma* subsp. *sororia* and its putative center of origin is in central and southern Mexico^16^. Phytolith data suggests the presence of a domesticated cucurbit, possibly *C. argyrosperma*, in the central Balsas River valley of Mexico as early as 8700 cal B.P.^17^. The earliest unambiguous archaeological specimen of *C. argyrosperma* is a ~ 5100 cal B.P. peduncle from the Ocampo caves in northeastern Mexico^11^. Other archaeological and archaeogenomic evidence reveals that following *C. argyrosperma*’s domestication, it diffused northeast of Mexico into the eastern and central portions of the United States before European contact^4,15,18,19^. For instance, *C. argyrosperma*’s seeds have been recovered from Late Mississippian contexts in Arkansas and it has been suggested that this cultivar was established in the region ~ 1310–623 cal B.P.^18^.

*Cucurbita maxima* was domesticated in the Amazonian tropical lowlands^10,20^. *C. ficifolia* and *C. moschata* were also likely domesticated in South America, but neither their wild ancestors nor centers of origin are currently well defined^10,16,20^. *C. moschata* appears to have arrived in Mexico from farther south as suggested by the identification of distinctive phytoliths dated between 9240 cal B.P. in Panama and desiccated macrobotanical remains dated to 6970 cal B.P. in the northern coast of Peru^7,21,22^.

Advanced molecular research has resolved the major phylogenetic relationships among squash and gourd species. In addition, archaeobotanical approaches can provide tangible evidence of these crops’ movements across space and changes over time. For example, systematic morphometric analysis of *C. pepo* seeds has documented diachronic changes in seed traits and size that can be used to differentiate between wild and domesticated subspecies of *C. pepo*. Because of a lack of similar studies in other cucurbit species, it is unknown whether similar morphological changes associated with a domestication syndrome can be traced. The use of morphometric seed characters (e.g., length, width, and thickness) is further complicated by significant size overlap between wild and domesticated varieties and by seed deformation caused by preservation conditions, cooking, and carbonization^23^. These issues contribute to a persistent lack of certainty when identifying cucurbit seeds from archaeological contexts. In this paper, we address the question of how human selection shaped domesticated *Cucurbita* and *Lagenaria* by conducting a quantitative analysis of desiccated seeds, rinds, and stems from the well-dated chronological sequence of El Gigante, a dry rock shelter located in south-central Honduras. We employ direct radiocarbon dating and a range of multivariate quantitative methods to identify possible changes in size and shape of seeds and rinds to distinguish between wild and domesticated cucurbits over time. The goal of this approach was to fundamentally improve our understanding of the tempo and mode of the domestication of these plant cultivars.

### El Gigante Rockshelter

**Figure 1:**
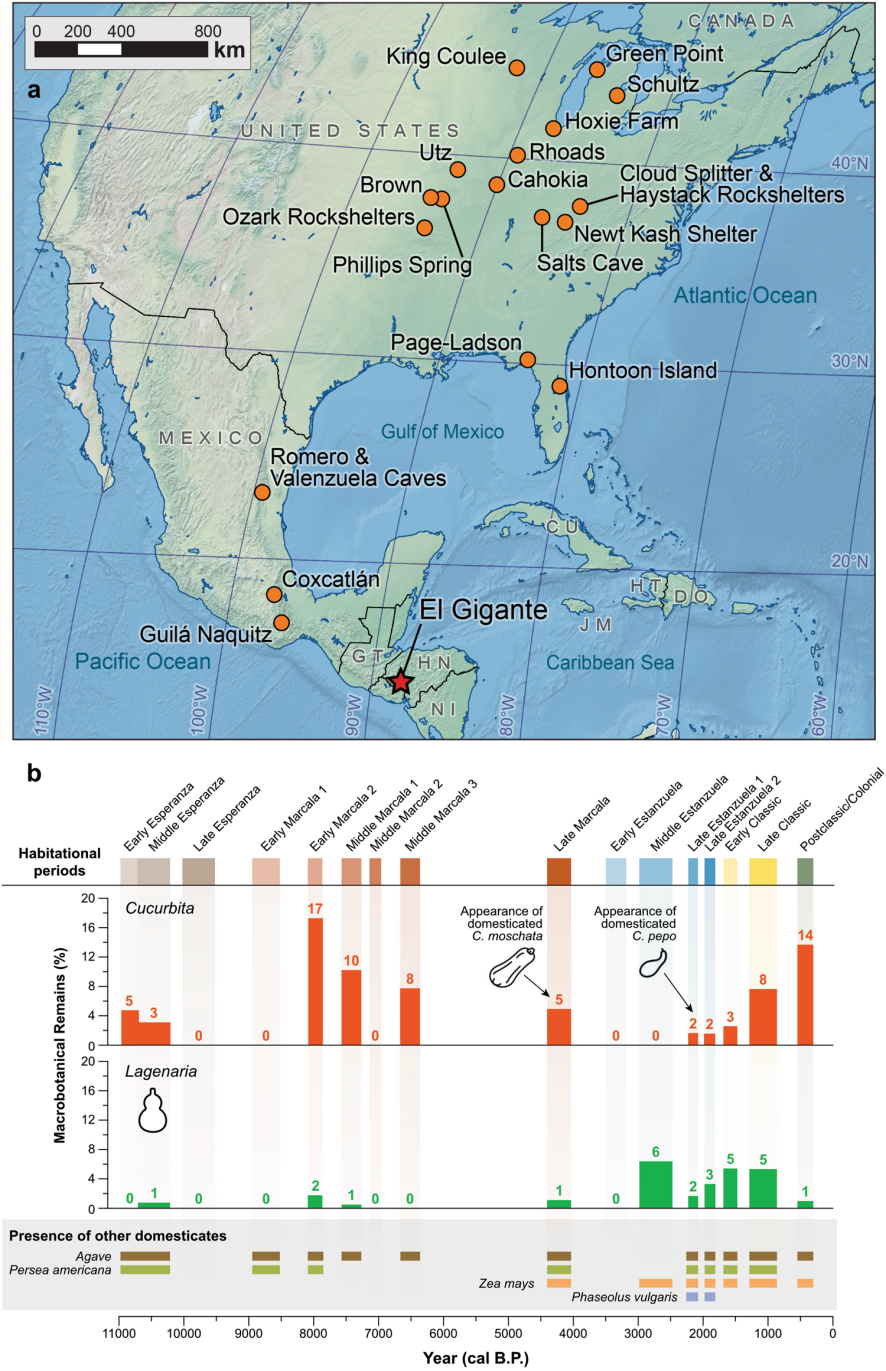
(**a**) Location of El Gigante Rockshelter and archeological sites with *Cucurbita* measured seeds used in the analysis. (**b**) Percentages of macrobotanical remains of *Cucurbita* and *Lagenaria* and other field crops from El Gigante Rockshelter, Honduras.

El Gigante (14° 13ʹ N 88° 03ʹ W, 1300 m above sea level) is a large rock shelter located along the western ridge of the Estanzuela River in the highlands of western Honduras (Fig. 1). The site was excavated in 2000 and 2001^24,25^. The occupation at the site spans great time depth with deposits dating from the Terminal Pleistocene through the Postclassic/Colonial periods. Bayesian chronological modeling of radiocarbon dates from the rock shelter suggest 16 phases of occupation^26^. The initial occupation took place during the Early and Middle Esperanza phases between 10,985–10,705 and 10,705–10,210 cal B.P., respectively, and was characterized by the presence of stemmed bifaces and avocado, squash and maguey remains. After a gap of 190 years, the Late Esperanza (10,020–9,520 cal B.P.) featured wide fluted projectile points as well as ciruela (*Spondias*), squash, maguey, and avocado remains^27^. The subsequent phase of occupation, Marcala, was episodic. The Early Archaic (Early Marcala 1: 8945–8520 cal B.P., Early Marcala 2: 8090–7865 cal B.P.) and Middle Archaic (Middle Marcala 1: 7570–7265 cal B.P., Middle Marcala 2: 7140–6960 cal B.P., and Middle Marcala 3: 6665–6365 cal B.P.) habitations are characterized by the abundance of ciruela, avocado, squash, and maguey remains. A long occupation hiatus took place between 6365 and 4440 cal B.P. The Late Marcala phase (4400–4025 cal B.P.) corresponding to the regional Late Archaic is characterized by the appearance of maize (*Zea mays*), which is found along with tree fruits, including ciruela and avocado as well as a low abundance of squash. Following a second hiatus of 530 years, the Early and Middle Formative occupation (Early Estanzuela phase: 3500–3185 cal B.P.; Middle Estanzuela: 2985–2470 cal B.P.) features the appearance of ceramics. The Late Formative occupation (Late Estanzuela phase 1: 2225–2080 cal B.P.; Late Estanzuela phase 2: 1980–1810 cal B.P.) contains large quantities of maize and the appearance of beans (*Phaseolus* spp.). During the Classic period (Early Classic: 1690–1475 cal B.P., Late Classic: 1295–865 cal B.P.), maize continues to constitute a main component of the assemblage, and avocados, squash, and wild fruits increase in abundance. The occupation of the rock shelter lasted through the Postclassic/Colonial eras (545–310 cal B.P.), periods when the site was rarely used. During the most recent occupations, acorns were common, but maize and gourds were rare in the assemblage.

## Results

### Direct radiocarbon dating

Direct accelerated mass spectrometry (AMS) radiocarbon (^14^C) dating of desiccated cucurbit rinds, stems, and seeds throughout the 11,000-year occupation of El Gigante has revealed a long history of cucurbit use by humans (Table 1). The earliest analyzed *Cucurbita* rinds date from the Esperanza phase and were directly AMS ^14^C dated to 11,090–10,720 (2σ; PSUAMS-8055) cal B.P. and 10,655–10,425 (2σ; PSUAMS-8251) cal B.P. Two Early Marcala-phase *Cucurbita* rinds dated to 8595–8430 (2σ; PSUAMS-8064) cal B.P. and 8020–7880 (2σ; PSUAMS-8056) cal B.P., while two Middle Marcala rinds dated to 6635–6485 (2σ; PSUAMS-8063) cal B.P. and 6495–6395 (2σ; PSUAMS-8062) cal B.P. One Late Marcala *Cucurbita moschata* stem was dated to 4145–3930 (2σ; PSUAMS-6129) cal B.P. We recovered *Cucurbita* seeds only from the Estanzuela (Formative) and Classic-period deposits. One *C. pepo* seed resulted in a date of 2305–2060 (2σ; PSUAMS-6128) cal B.P., and a *C. moschata* seed yielded a date of 1245–1065 (2σ; PSUAMS-6126) cal B.P.

**Table 1:** Direct radiocarbon dates of desiccated cucurbits samples from El Gigante Rockshelter, Honduras.

Lab code	Taxon	Element	^14^C age and error	Median cal age (B.P.)	2 Sigma cal age (B.P.)	Provenience	Cultural phase
PSUAMS-8058	*Lagenaria*	Rind	9595 ± 30	10,935	11,150–10,765	Unit 18, Level 39	Early Esperanza
PSUAMS-8055	*Cucurbita*	Rind	9560 ± 35	10,915	11,090–10,720	Unit 18, Level 36	Early Esperanza
PSUAMS-8054	*Cucurbita*	Rind	9540 ± 30	10,900	11,075–10,700	Unit 18, Level 35	Early and Middle Esperanza
PSUAMS-8057	*Cucurbita*	Rind	9510 ± 30	10,850	11,070–10,660	Unit 18, Level 39	Early Esperanza
PSUAMS-8052	*Cucurbita*	Rind	9370 ± 30	10,595	10,690–7940	Unit 3, Level 27	Middle Esperanza
PSUAMS-8251	*Cucurbita*	Rind	9335 ± 30	10,543	1054–10,427	Unit 18, Level 34	Middle Esperanza
PSUAMS-8064	*Cucurbita*	Rind	7745 ± 25	8515	8595–8430	Unit 18, Level 31	Early Marcala 2
PSUAMS-5381	*Cucurbita*	Rind	7205 ± 30	8005	8165–7935	Unit 18, Level 32b	Early Marcala 2
PSUAMS-5382	*Cucurbita*	Rind	7205 ± 30	8005	8165–7935	Unit 18, Level 33a	Middle Esperanza
PSUAMS-8053	*Cucurbita*	Rind	7175 ± 25	7985	8025–7940	Unit 18, Level 33b	Early Marcala 2
PSUAMS-8059	*Lagenaria*	Rind	7170 ± 25	7985	8020–7935	Unit 18, Level 33b	Early Marcala 2
PSUAMS-8056	*Cucurbita*	Rind	7155 ± 30	7975	8020–7880	Unit 18, Level 37	Early Marcala 2
PSUAMS-1786	*Cucurbita*	Rind	6540 ± 30	7455	7565–7355	XXIV, East wall	Middle Marcala 3
PSUAMS-8063	*Cucurbita*	Rind	5755 ± 20	6555	6635–6485	Unit 18, Level 24	Middle Marcala 1
PSUAMS-8062	*Cucurbita*	Rind	5670 ± 20	6445	6495–6395	Unit 18, Level 19	Late Marcala
PSUAMS-6127	*Lagenaria*	Rind	3835 ± 20	4230	4390–4145	Unit 18, Level 26	Middle Marcala 1
PSUAMS-8061	*Lagenaria*	Rind	3735 ± 20	4080	4155–3985	Unit 18, Level 18	Late Marcala
PSUAMS-6129	*Cucurbita moschata*	Peduncle	3695 ± 20	4035	4145–3930	Unit 19, Level 21	Late Marcala
PSUAMS-6128	*Cucurbita pepo*	Seed	2165 ± 20	2195	2305–2060	Unit 6, level 13	Mostly Late Estanzuela 1
Beta-316169	*Lagenaria*	Rind	2110 ± 30	2075	2290–1995	Unit 18, Level 13b	Late Estanzuela 1
PSUAMS-8060	*Lagenaria*	Rind	2105 ± 15	2065	2120–2000	Unit 18, Level 19	Late Marcala
PSUAMS-8454	*Lagenaria*	Worked rind	2100 ± 15	2058	2115–2001	Unit 18, Level 9	Late Estanzuela 1
UCIAMS-112159	*Cucurbita*	Seed	2090 ± 20	2050	2120–1995	Unit 18, Level 3B	Early Classic, slightly disturbed
UCIAMS-112158	*Cucurbita*	Seed	2020 ± 20	1960	2000–1885	Unit 10, Level 16	Mixed due to Burial
PSUAMS-8451	*Lagenaria*	Worked rind	1765 ± 15	1655	1709–1611	Unit 8, Level 6	Disturbed; Early Classic-Colonial Mix
PSUAMS-8453	*Lagenaria*	Worked rind	1605 ± 15	1470	1532–1413	Unit 14, Level 2	Disturbed
PSUAMS-6126	*Cucurbita moschata*	Seed	1225 ± 20	1145	1245–1065	Unit 4, Level 11	Disturbed
PSUAMS-8450	*Lagenaria*	Worked rind	360 ± 15	404	490–320	Unit 4, Level 2	Postclassic/Colonial
PSUAMS-8452	*Lagenaria*	Worked rind	310 ± 15	383	439–308	Unit 11, Level 2	Postclassic/Colonial

*Lageneria* remains were found from the Esperanza to the Colonial occupation phases. The earliest *Lagenaria* rind, from the Early Esperanza phase, was directly dated to 11,150–10,765 (2σ; PSUAMS-8058) cal B.P. Rinds from Archaic deposits returned results of 8020–7935 (2σ; PSUAMS-8059; Early Marcala 2) cal B.P. and 4155–3985 (2σ; PSUAMS-8061; Late Marcala) cal B.P. Late Estanzuela *Lagenaria* rinds yielded dates between 2290 and 2000 (2σ; Beta-316169, PSUAMS-8060 and PSUAMS-8454) cal B.P.

### Rind fragments

Based on their morphological attributes (14), we identified a total of 448 rind fragments (weighing 16.5 g) of *Cucurbita* and 387 rind fragments (weighing 56.3 g) of *Lagenaria*. *Cucurbita* rind fragments were found in all occupation phases (i.e., Esperanza, Marcala, Estanzuela, Classic, and Postclassic/Colonial). The vast majority of *Lagenaria* rinds were recovered from contexts associated with the Estanzuela (38%) and Classic (53%) occupation phases, while few rinds were recovered from the Esperanza (6%) and Marcala (2%) phases.

**Figure 2:**
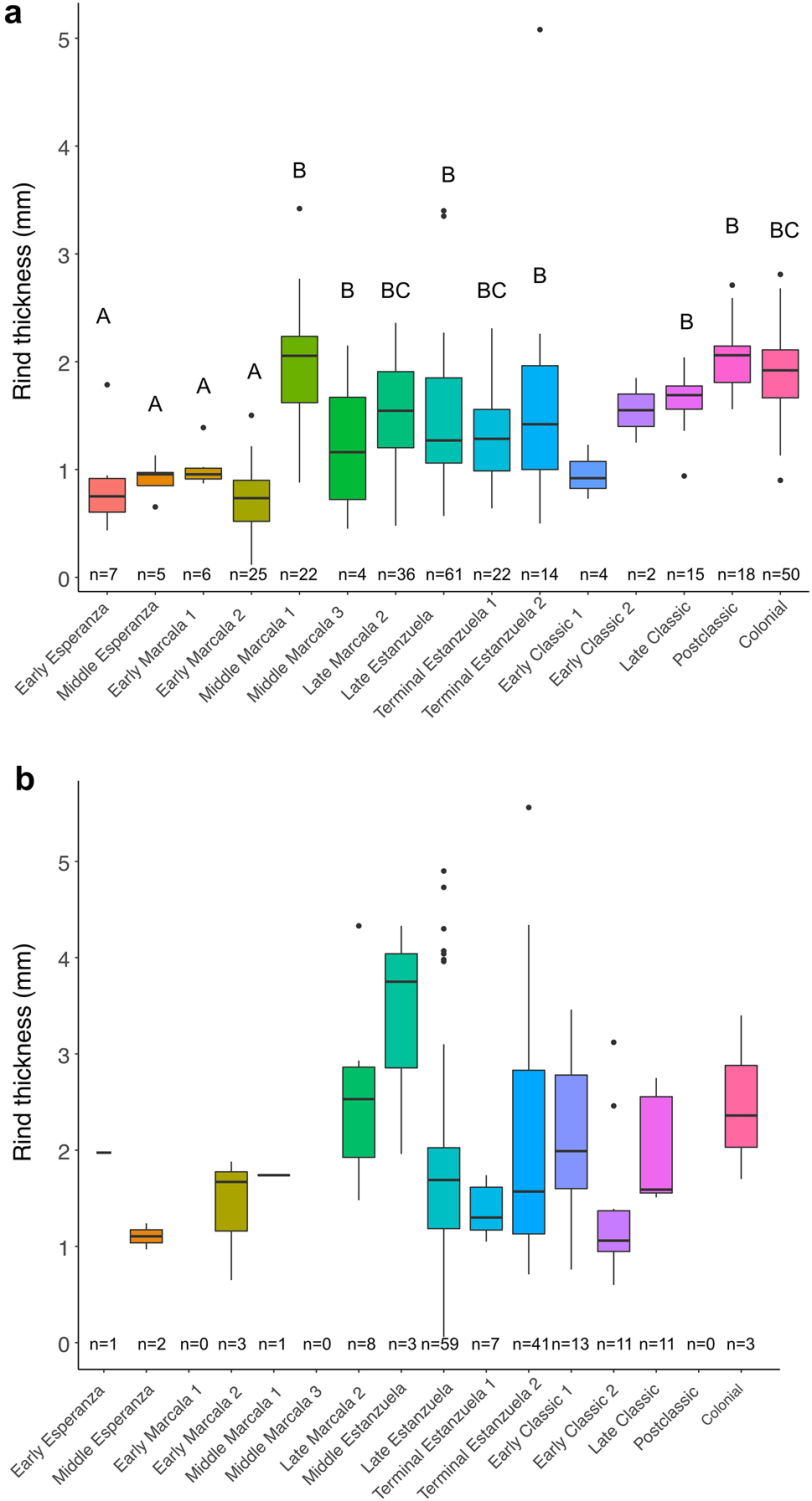
Variation of rind thickness of (**a**) *Cucurbita* and (**b**) *Lagenaria* recovered across occupational phases from El Gigante Rockshelter, Honduras. Different letters indicate significant differences (*P* < 0.05). The sample size is indicated below each boxplot.

**Figure 3:**
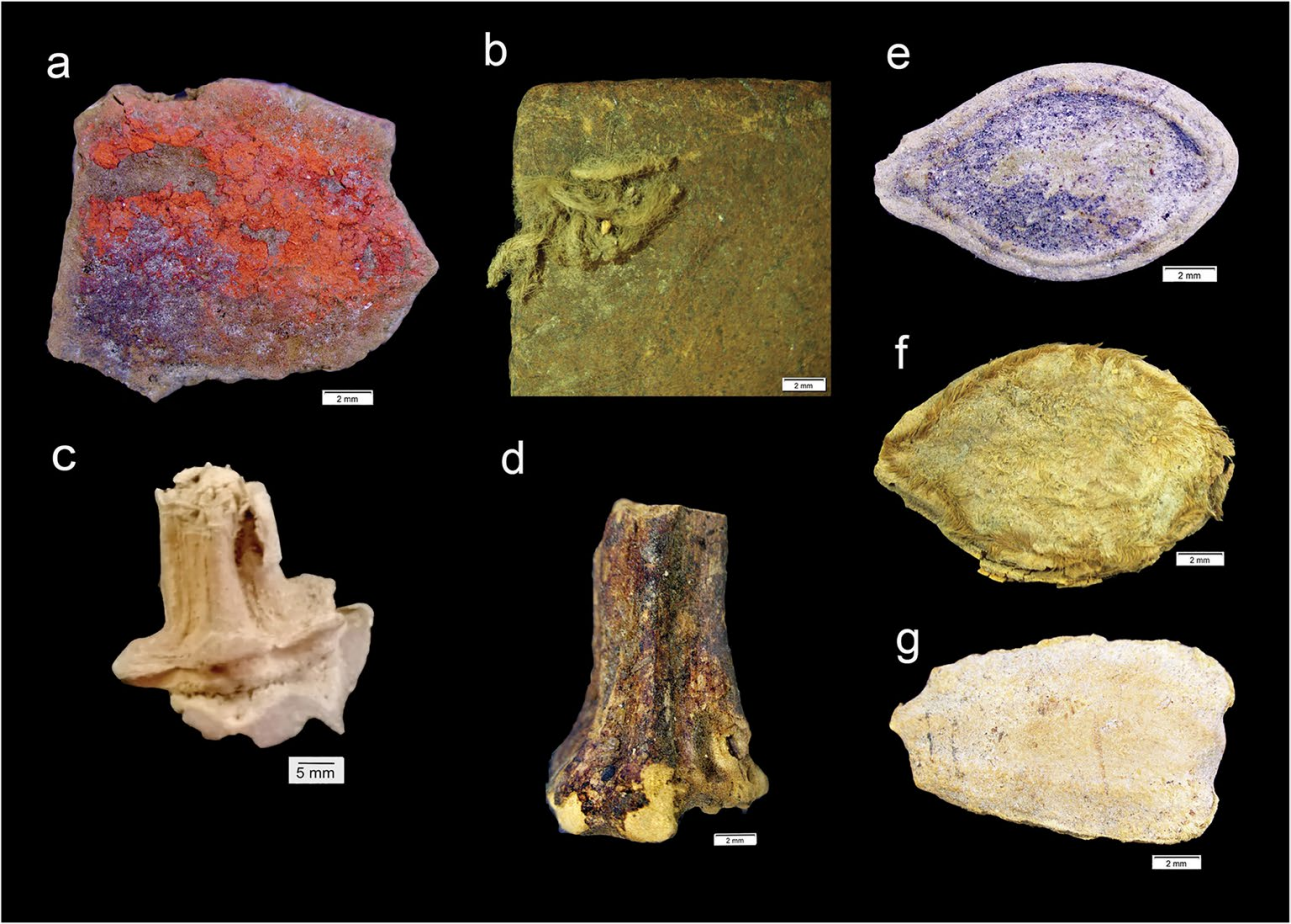
Cucurbit desiccated remains recovered from El Gigante Rockshelter, Honduras. (**a**) *Cucurbita* rind stained with red pigment. (**b**) Polished *Lageneria* rind with attached rope. (**c**) *Cucurbita pepo* peduncle. (**d**) *Cucurbita moschata* peduncle (PSUAMS-6129, 4035 cal B.P.). (**e**) *Lagenaria siceraria* seed. (**f**) *C. pepo* seed. (**g**) *C. moschata* seed.

Thickness of *Cucurbita* rind fragments increase significantly over time (F_12,151_ = 11.65, P < 0.0001) (Fig. 2a). The thickness of *Lagenaria* rinds, however, showed little variation over time (F_14,284_ = 1.53, P = 0.12) (Fig. 2b). However, given the unequal sample size between occupation phases, results should be interpreted with caution. Evidence of post-harvest modifications in rinds of *Cucurbita* is notable, particularly thermal alteration during the Esperanza (10,990–9500 year B.P.) and Marcala (8950–4020 year B.P.) phases. Specifically, a large proportion of *Cucurbita* rind fragments recovered from the Esperanza (50%) and Marcala (66.7%) phases were burned. In contrast, only a few of the rinds from the later Estanzuela (3500–1810 year B.P.; 5%) and Postclassic/Colonial (545–310 year B.P.; 11.1%) phases showed evidence of burning. One small *Cucurbita* rind fragment recovered from Unit 12, Level 4 was colored red (Fig. 3a). We found that only 3.6% of the rinds of *Lagenaria* had evidence of abrasion. A few *Lagenaria* rinds (n = 5) were polished on one side. These rinds yielded AMS dates between 2120 and 320 cal B.P. (2σ; PSUAMS-8450, -8451, -8452, -8453 and -8454). For example, one rind fragment recovered from Unit 11, level 2 was polished on one side with evidence of deliberate holes and included attached to fragments of cordage (Fig. 3b).

### Peduncles

A total of 16 *Cucurbita* peduncles was recovered, of which seven were identified as *C. pepo* and seven as *C. moschata* (following 14, 15, 49, 50). *C. pepo* stems exhibited seven to nine major ridges that had a mean basal diameter of 19.7 ± 0.5 mm (Fig. 3c). *C. moschata* peduncles were characterized by the presence of five major ridges, a pentagonal base (Fig. 3d), and a mean basal diameter of 13.9 ± 1.7 mm.

### Seeds

A total of 71 ancient cucurbit seeds were recovered from El Gigante. Most of these seeds originate from Late Estanzuela, later, or mixed contexts. Based on their external morphology, we identified these seeds as *C. pepo* (n = 48), *C.* cf. *pepo* (n = 3), *C. moschata* (n = 5), *Cucurbita* sp. (n = 6), and *Lagenaria* (n = 9) (Fig. 3f,g).

**Figure 4:**
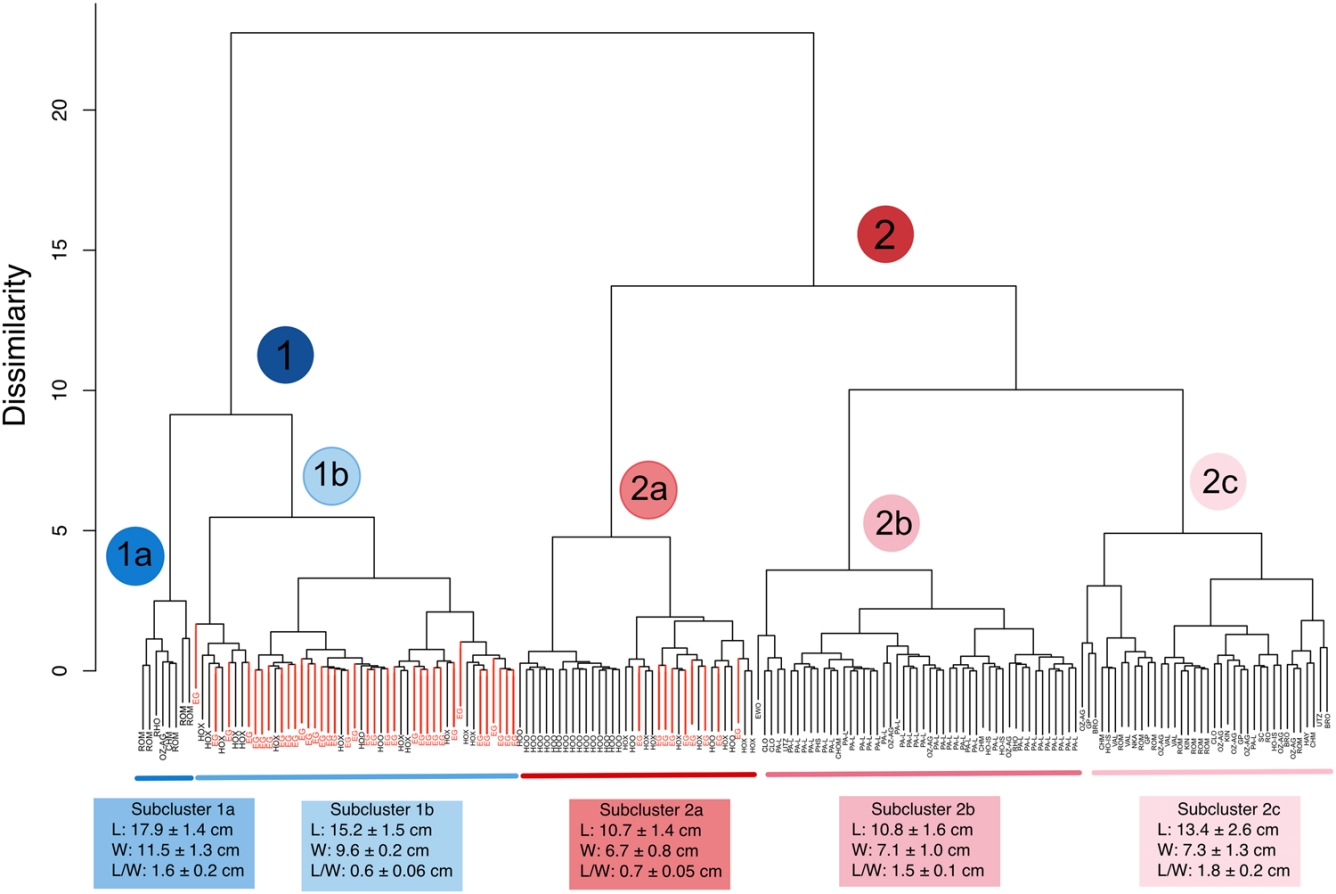
Hierarchical cluster analysis of paleontological and archaeological *Cucurbita pepo* seeds using Ward’s agglomerative hierarchical classification based on maximum length, maximum width, and length/width ratio. Seeds recovered from El Gigante (EG) are highlighted in red. *BRO* Brown^28^, *CHM* Mount 51 Cahokia—Chmurny’s type 1^29^, *CLO* Cloudsplitter^30^, *GP* Green Point^31^, *HAY* Haystack^30^, *HOO* Hoonton Island^5^, *HOX* Hoxie site^32^, *KIN* King Coulee^33^, *NKA* Newt Kash Hollow^30^, *OZ-AG* Ozarks—Agnew Small, Beaver Pond and Whitney Bluff^19^, *PA-L* Page-Ladson, *PHS* Phillips Spring^34,35^, *RHO* Rhoads^36^, *RO* Rogers^30^, *ROM* Romeros cave^12^, *SAG* Saginaw^37^, *SC* Salts Cave^34^, *UTZ* Utz^38^, *VAL* Valenzuelas Cave^12^.

A hierarchical cluster analysis comparing previously published paleontological and archaeological *C. pepo* seeds from sites throughout Central and North America (Fig. 1) generated two main morphological groups, which can be subdivided in five additional subgroups (Fig. 4). Cluster 1 includes the largest seeds. Subcluster 1a includes the longest (17.9 ± 1.4 cm) and widest seeds (11.5 ± 1.3 cm), while subcluster 1b comprises slightly shorter (15.2 ± 1.5 cm) and narrower (9.6 ± 0.2 cm) seeds. In contrast, cluster 2 includes the smallest seeds, from which subcluster 2a (length: 10.7 ± 1.4 cm, width: 6.7 ± 0.8 cm) includes the shortest and narrowest seeds in comparison to subcluster 2b (length: 10.8 ± 1.6 cm, width: 7.1 ± 1.0 cm) and subcluster 2c (length: 13.4 ± 2.6 cm, width: 7.3 ± 1.3 cm). The El Gigante archaeological *C. pepo* seeds fell either in subcluster 1b (n = 39) or subcluster 2a (n = 9) suggesting the presence of at least two phenotypes (Supplementary Tables S2, S3).

**Figure 5:**
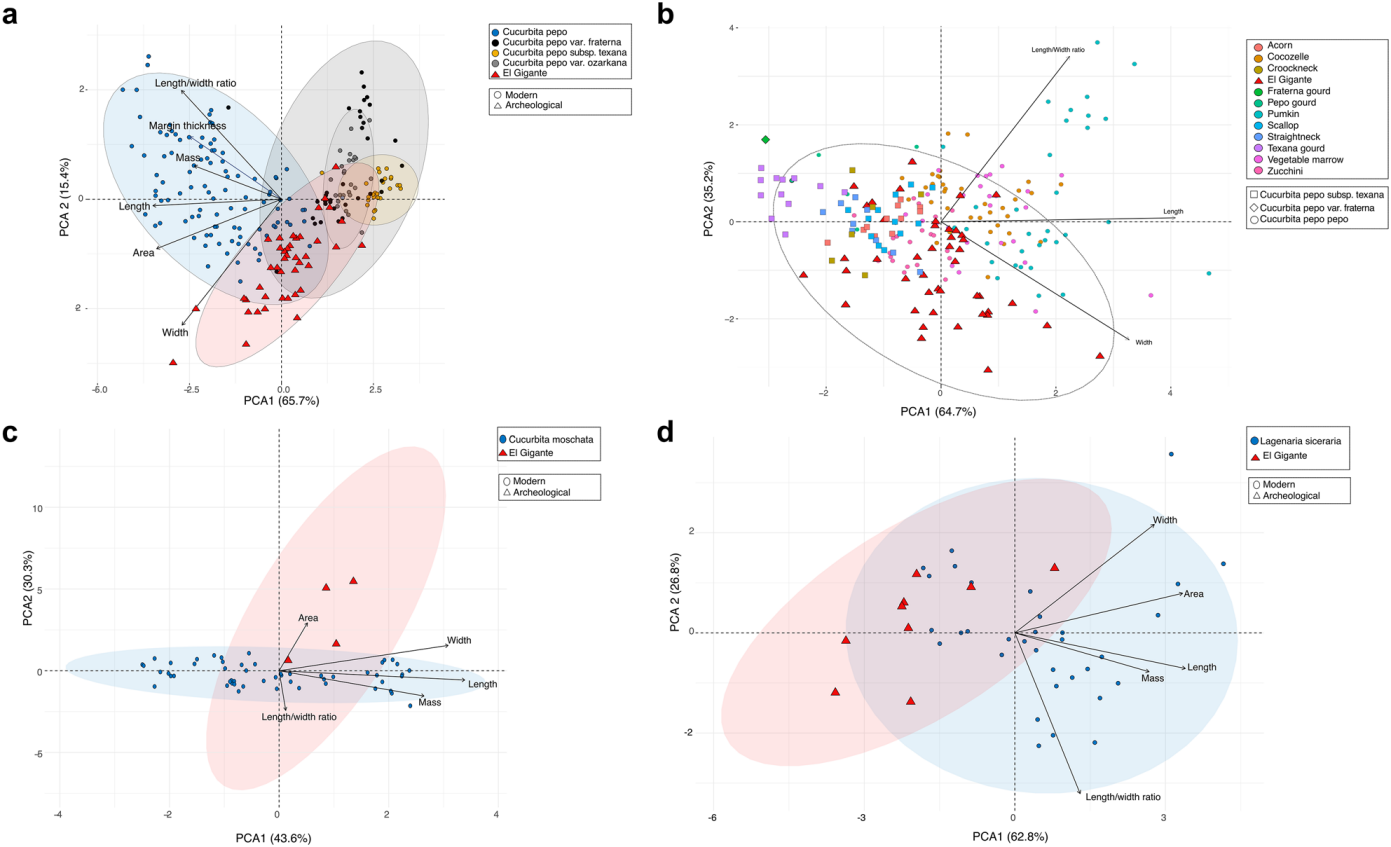
Principal component analysis biplots comparing size of archaeological seeds from El Gigante Rockshelter with modern seeds in (**a**) *Cucurbita pepo*; (**b**) *C. pepo* varieties^39^; (**c**) *Cucurbita moschata*; and (**d**) *Lagenaria siceraria* based on morphometric measurements.

To better understand the variability in the archaeological assemblage, we compared the archaeological *C. pepo* seeds from El Gigante to a dataset of modern wild and domesticated *C. pepo* seeds that we measured (Supplementary Table S1). Principle component analysis (PCA) of morphometric traits allow us to separate modern variants of domesticated *C. pepo* seeds from those of wild *C. pepo* (*C. pepo* var*. fraterna*, *C. pepo* subsp*. texana* and *C. pepo* var. *ozarkana*) (Fig. 5a). The first two components collectively explain 78% of the variance in the data (61.5% and 16.8%, sequentially). Principal component 1 discriminated between total seed length and length to width ratio, while principal component 2 separated seeds by maximum width and seed margin width. Measurements of archaeological seeds recovered from El Gigante demonstrate that most cucurbit seeds are comparable in size to modern domesticated *C. pepo* seeds. Only six of the El Gigante seeds are sufficiently small to fall within the confidence interval ranges of wild *C. pepo* seeds and specifically, the varieties *C. pepo* var*. ozarkana* and *C. pepo* var*. fraterna.*

To evaluate if the ancient *C. pepo* seeds recovered from El Gigante are similar to modern varieties of domesticated and wild *C. pepo,* we performed a second PCA using the cucurbit cultivar measurement data reported in Ref.^40^. Principal component 1 (63% of total variance) discriminated among seeds by total length and maximum width whereas principal component 2 (35.2% of total variance) separated seeds according to their length to width ratio (Fig. 5b). The biplot shows high variability within the El Gigante archaeological seeds and the fact that the first two components captured 98% of the variance attests to the strength of the relationships among the cases and variables. Ten archaeological seeds were morphometrically similar to modern acorn, scallop and crookneck varieties of wild *C. pepo* subsp. *texana.* Sixteen seeds overlap with modern seeds of cocozelle, vegetable marrow and zucchini varieties of *C. pepo* subsp. *pepo*. Finally, 17 of the archaeological seeds are not associated with any variety of modern *C. pepo* in particular.

A multivariate analysis shows that only one archaeological seed of *C. moschata* from El Gigante is morphometrically similar to its modern equivalent (Fig. 5c). Principal component 1, which accounts for 43.6% of the variance, separates seeds by total length, maximum width, and mass. Principal component 2, accounting for 30.3% of the variance, separates seeds by surface area and length to width ratio. Three of four archaeological seeds fell outside the confidence intervals of modern *C. moschata.* Archaeological seeds have low principal component 1 scores, which are reflective of small seeds (small length and small width), but high principal component 2 scores, indicating that they have a broader surface area than most of the modern seeds.

A final PCA comparing ancient and modern *L. siceraria* seeds shows that the first component (62.8% of the variance) differentiates seed surface area, total length, and mass. The second component (26.8% of the variance) is related to maximum width and length to width ratio (Fig. 5d). Most of the El Gigante *Lagenaria* seeds are distributed along the left side of the first component, showing their smaller size in relation to modern seeds.

**Figure 6:**
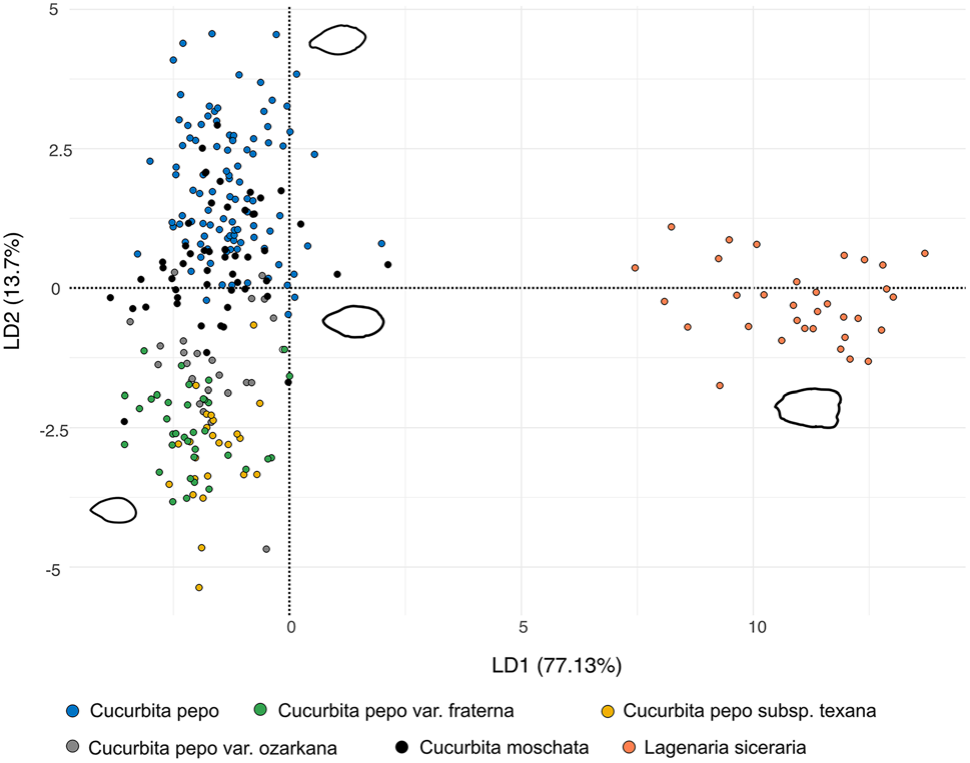
Linear discriminant analysis (LDA) biplot of axes 1 and 2 (77.1% and 13.7% of the total variance, respectively) showing the morphological gradient of modern *Cucurbita* seeds in relation to *Lagenaria siceraria* according to the elliptic Fourier transform (EFT) method. Seed outlines represent approximate seed shape.

To assess shape variation, we calculated elliptical Fourier descriptors (EFDs) (Fig. 6). The results largely show that *Cucurbita* species are clearly discriminated from *Lagenaria siceraria* on the LDA1-LDA2 biplot (94% of the total variation). Furthermore, the LDA discriminates the seeds of domesticated *C. pepo* from the wild *C. pepo* species (*C. pepo* var*. fraterna*, *C. pepo* var. *ozarkana*, and *C. pepo* subsp. *texana*). It is notable, however, that there is significant overlap in seed shape between *C. pepo* and *C. moschata* and between *C. pepo* var. *ozarkana* and *C. moschata*.

The geometric morphometric analysis indicates that the shape of the archaeological gourd and squash seeds from El Gigante vary considerably. Of the 21 archaeological seeds morphologically identified as *C. pepo*, the elliptical Fourier transform (EFT) method correctly classified only three specimens as *C. pepo*. The remaining archaeological seeds were allocated to the wild *C. pepo* group (seven as *C. pepo* var. *ozarkana*, eight as *C. pepo* var. *texana*, and three as *C. pepo* var. *fraterna*). Only one of the archaeological *C. moschata* seeds was correctly classified as *C. moschata*, while the other two seeds were attributed to the *C. pepo* group. Finally, most of the archaeological *Lagenaria* seeds (n = 5) were correctly classified as *L. siceraria*, but three were assigned as *C. pepo* and one as *C. pepo* var. *ozarkana*. In light of the variability inherent in cucurbit seeds, this method has limited power to add to morphological classification in this case.

## Discussion

The chronology, size, and geometric morphometric analyses of *Cucurbita* and *Lagenaria* macrobotanical remains from El Gigante Rockshelter reveal intriguing patterns related to human reliance, use, and cultivation of cucurbits for food and technology during the last ~ 11,000 years. Key changes in plant selection and use correspond with broader subsistence and settlement changes spanning the Esperanza and Marcala phases, a time span that represents the long transition from plant foraging to low-level cultivation.

Ancient people inhabiting the area around El Gigante consistently used *L. siceraria* and *Cucurbita* through the entire sequence of occupation of the site. Initially, *Cucurbita* rinds likely gathered from the wild were significantly thinner than rinds recovered from subsequent periods when cultivated varieties were probably utilized. Research has shown that an increase in rind thickness is associated with the presence of domesticated squash in contrast to the brittle rinds of wild squash forms^12^. El Gigante *Cucurbita* rinds recovered from the earliest occupation levels were < 2 mm thick, suggesting that humans used wild *Cucurbita* species during this time. In contrast, *Cucurbita* rind thickness increases during the Late Archaic (2.5 mm, range 0.5–5.1), indicating the introduction of domesticated *Cucurbita* to the region ~ 4000 years ago. A similar change in rind thickness over time has been documented in the Guilá Naquitz cave, where *Cucurbita* rinds showed an increase in thickness from 0.84 mm (range 0.5–1.6 mm) to 1.15 mm (range 0.5–2.0 mm) between ca. 10,500 and 8500 years ago^12^. In contrast, rinds of *C. maxima* recovered from the Pampa Grande archaeological site in Argentina show high variation in thickness through time (mean 4.09 mm, range 2.08–6.64 mm), suggesting that different varieties were cultivated and managed for different purposes (e.g., some as food and others as containers)^41,42^.

Given that a large percentage of *Cucurbita* rinds from these earlier occupations were burned, it seems likely that mobile hunter-gatherers used some cucurbits as cooking containers. We also suggest that some of these early, thinner cucurbits were roasted directly in proximity to cooking fires for consumption, potentially focusing on the squashes that had a lower concentration of bitter cucurbitacin than the rest of the fruits. In contrast, likely cultivated *Cucurbita* with more durable and thicker rinds could have been used to store food or water. The recovery of a *Cucurbita* rind stained with red pigment suggests that squash containers also may have been decorated vessels^42,43^.

The recovery of *Lagenaria* rinds and seeds spanning the occupation of El Gigante Rockshelter provides further evidence that gourds were used consistently during the early Holocene in Mesoamerica. AMS dating of a *Lagenaria* rind recovered from Unit 18, level 39 corresponding to the early Esperanza phase produced a date of 10,935 cal B.P. (11,150–10,765 cal B.P.). The date is considerably older than the most ancient specimens of bottle gourd previously reported, which were from Guilá Naquitz, Oaxaca (10,021 ± 105 cal B.P.; 10,198–9781 cal year BP) and Little Salt Spring, Florida (10,015 cal B.P.)^8,44^. *Lagenaria* was likely one of the first field crops in El Gigante and the light-weight fruits were used as containers even after ceramic technology was introduced to the region during the Early Formative period (~ 3600 cal B.P.).

*Lagenaria* rinds show little change in thickness over time. It is notable that El Gigante *Lagenaria* rinds exhibit a thickness (1.9 ± 1 mm) that overlaps with rinds of wild African *L. siceraria* (< 2.0 mm)^3^. El Gigante rinds are also consistently thinner than those recovered from other archaeological contexts in the Americas, including the Ocampo (3.2 mm), the Guilá Naquitz (4.6 mm), and the Coxcatlán (3.8 mm) caves in Mexico, and Ancón (4.8 mm) in Peru^3^. The domestication status of the recovered *Lagenaria* remains to be assessed in detail due to the absence of morphological characterizations in wild and domesticated *Lagenaria* species. These results highlight the importance of conducting further comparative studies of modern and archaeological plant remains alongside paleogenomic analyses to assess the domestication syndrome in *Lagenaria* and its possible center of domestication in south-central Mesoamerica.

Morphometric studies of variation in seed size improve our understanding of how domestication shaped plant diversity^45^. Refined methods of morphometric analysis are critical if we are to fully understand the processes involved in the domestication and spread of these cultigens. Geometric morphometric analysis is a complementary approach that can be used to assess changes in seed shape that may be associated with domestication. This method has been useful in the analysis of several crop species, including olives, grapes, barley, and date palms^46–49^. For instance, seed morphology has been used to distinguish between pips of wild and domesticated grape (*Vitis vinifera*) varieties in Italy^50^. Our results indicate that domesticated *C. pepo* show discernable differentiation in seed size and shape from wild species (*C. pepo* var. *fraterna*, *C. pepo* subsp. *texana*, and *C. pepo* var. *ozarkana*)*.* Furthermore, distinct groups of *C. pepo* and *C. pepo* visualized with PCA and EFDs confirm existing species distinctions based on morphological data. We argue that the high level of phenotypic variability in cultivated *C. pepo* is a consequence of varietal diversification and reflects the long-term history of cultivation associated with the selection of specific morphological traits, such as fruit size and shape. These morphological differences may be explained by divergent selection pressures. For instance, wild *C. pepo* species were subject to environmental constraints that seemed to have minimized seed size, while domesticated *C. pepo* were subject to ongoing human selection related to cultivation practices that shaped particular phenotypes.

In contrast to fruit size, it is likely that seed shape was not a direct target of human selection in *C. pepo*. The evolutionary role of seed shape in cucurbits is not well understood, but shape changes could be related to a pleiotropic effect that determines seed persistence in the soil, seed germination and dispersal^51^. Some empirical studies have shown that long-term persistent species tend to have spherical seeds, while transient species produce flattened and/or elongated seeds^52,53^. Genetic drift associated with the restricted gene pool of breeding can also have essentially random implications for non-selected traits such as seed shape.

The shape analysis was not able to differentiate seeds of *C. moschata* from subspecies of *C. pepo.* Modern seeds of *C. moschata* showed a high overlap with both the domesticated *C. pepo* and the wild *C. pepo* var. *ozarkana*. The results expose the limitations of using shape analysis for identifying *C. moschata* seeds and highlights the importance of including other qualitative characters for identification.

Multivariate morphometric analyses show that most of the *C. pepo* seeds from El Gigante fall within the size parameters defined for modern types of domesticated *C. pepo* seeds. Paris and Nerson^39^ have also shown a relationship between seed size and fruit size in *C. pepo*. Our analysis suggests that some of the archaeological seeds are similar to domesticated present-day varieties, including the cocozelle and zucchini varieties, which are characterized by long and uniformly cylindrical fruits, and the vegetable marrow variety, which is distinguished by short round fruits with a slightly broad apex^39^.

The seeds of *C. pepo* that are similar in size to wild *C. pepo* subspecies could have been the product of genetic exchange between crops and wild cucurbit species in the area. Gene flow and crop-to-wild hybridization have been reported to be common in *C. pepo* due to pollen transfer between plants by bees^40,54^, and hybrid plants have been shown to produce highly variable fruit sizes^54^.

In contrast to the morphometric results, shape analysis via linear discriminant analysis (LDA) allocated most of the El Gigante *C. pepo* seeds to wild forms. These results could suggest the presence of an unknown autochthonous variety or a separate subspecies that has since become extinct, for instance the as-yet-unknown wild progenitor of *C. pepo* ssp. *pepo* cultivated types. They also suggest that the region in which El Gigante is located may have been an important setting of squash diversification. A molecular analysis could help to better address squash diversity via aDNA analysis. However, the difficulty of differentiating wild from domesticated seeds in archaeological contexts could be associated with the preservation of the seed remains. Even though this analysis only included complete and well-preserved seeds, it is possible that the desiccation process might have altered the shape of some seeds.

## Conclusions

The archaeobotanical record from El Gigante Rockshelter suggests early indigenous foragers utilized wild cucurbits for generations before the introduction of cultivated varieties. Identifications of maize pollen and microcharcoal particles recovered from wetlands in Lago Yojoa ~ 5400 cal B.P. and in the Copan Valley ~ 4300 cal B.P. suggests that the adoption of farming in Mesoamerica was a relatively early and protracted process^55,56^. In correspondence with these studies, domesticated butternut squash seems to have been initially introduced in El Gigante during the Late Marcala phase, between 4400 and 4025 cal B.P., yet the utilization of wild cucurbits and other tropical plants persisted over time. In fact, as indigenous farmers began to substantially modify their surrounding tropical ecosystems, they were able to successfully incorporate domesticated crops such as maize and squash while still relying on and likely managing other resources such as tree crops (e.g., *Persea americana*, *Pouteria* spp., and *Spondias* spp.) for their subsistence^26^.

The significance of El Gigante lies in both the length of its chronology and its extensive well-preserved plant assemblage. The rock shelter thus has great potential to both broaden and reshape our understanding of long-term human adaptations to domestication and early agriculture. The analytical techniques we used (digital morphometrics, multivariate statistics, and outline analysis based on EFDs) proved to be powerful tools for assessing variation in rind thickness and seed size and shape. Indeed, geometric morphometric approaches combined with traditional biometric methods of length and width measurements have allowed us to distinguish among cucurbit species, specifically domesticated *C. pepo*, wild *C. pepo*, and *Lagenaria*. We note, however, that the classification accuracy of *C. moschata* from *C. pepo* based on EFDs is more limited compared to qualitative morphological attributes.

The study of the plant remains recovered from El Gigante Rockshelter substantially contributes to improving our understanding of Indigenous human-plant coevolution and the dispersal of key cultigens^57,58^. As part of broader research, this paper presents an important piece of the tropical Americas domestication puzzle.

## Materials and methods

### Recovery of macrobotanical remains

El Gigante is one of six dry rock shelters in Mesoamerica that contain well-preserved macrobotanical assemblages that include both desiccated and carbonized remains^57^. The plant remains analyzed in this study were either hand-collected in situ during excavations or recovered through sieving (mesh size 4.5 mm and 500 μm) sediment from Unit 18. Well-preserved cucurbit (*Cucurbita* and *Lagenaria*) remains in the assemblage include rinds, peduncles, and seeds. *Cucurbita* rinds (n = 448) were distinguished from *Lagenaria* rinds (n = 387) based on diagnostic cross-section cell structure (Supplementary Fig. S1). *Cucurbita* rinds exhibit isodiametric cells with a regular configuration, a feature that enabled us to distinguish them from *Lagenaria* rinds^12^. *Cucurbita* peduncles were identified at the species level based on the shape of the peduncle. Wild *Cucurbita* presents relatively small peduncles (< 13 mm in diameter) with circular outline; domesticated *C. pepo* peduncles are relatively bigger (> 14 mm in diameter) and are characterized by the presence of alternating ten major bridges with an angular or pentagonal outline at the fruit attachment, while *C. moschata* peduncle presents five ridges, with regular furrows and flaring at the base^12,13,59,60^.

### Direct radiocarbon dating

To further verify the age of the cucurbit assemblage, we conducted direct AMS ^14^C dating of 28 samples, including some of the earliest specimens in the assemblage. Samples were pretreated using conventional acid–base–acid methods and subsequently combusted, graphitized, and measured at the accelerated mass spectrometer of the Pennsylvania State University Radiocarbon Laboratory (PSUAMS). In addition, direct ^14^C dating of 310 additional (non-cucurbit) plant specimens allowed us to confidently assign the majority of cucurbit specimens to clarify chronological phases^26^.

### Modern seed material

To assess differences in seed shape and size, we relied on modern seeds from the USDA Germplasm Resources Information Network (GRIN). Samples included accessions from wild *Cucurbita* species (*C. pepo* subsp. *texana* [n = 25], *C. pepo* var. *ozarkana* [n = 25], and C. *pepo* var. *fraterna* [n = 35]) and domesticated species (*C. pepo* [n = 100], C. *moschata* [n = 48], and *L. siceraria* [n = 33]) originating from Mexico, Guatemala, and the United States (Supplementary Table S4). We also relied on previously published measurements of additional varieties of domesticated subspecies and cultivar groups of *C. pepo*^39^.

### Seed size and shape analysis

Digital images of the modern and archaeological seed samples were acquired using a digital camera attached to an Olympus SZX16 microscope with a 300-dpi resolution. Modern and archaeological seeds were photographed dorsally. Seeds were not photographed laterally because most of the archaeological seed coats were empty, resulting in lateral shape distortion. Digital images of the seeds were processed and measured using ImageJ v. 1.42^61^. The allometric characteristics of maximum length, maximum width, border thickness, and area were recorded in millimeters. Seeds were also weighed using a digital scale with a measuring error of 0.01 g. The full measurement datasets are included in Supplementary Tables S1 and S2.

Individual digital pictures were converted to binary images (i.e., seed image on a black background) in ImageJ. Each binary image was converted into chain code along the perimeter of each seed to create a harmonic series using SHAPE v. 1.3^62^. Seed shape was quantified using outline analysis based on EFDs to assess for differences in overall seed shape within species. Chain code contours were posteriorly converted to normalized EFDs for Fourier analysis.

### Data analysis

To assess differences in rind thickness between phases of occupation, we performed a one-way ANOVA for *Lagenaria* and *Cucurbita*, respectively. Differences in seed size between modern and archaeological seeds of *C. pepo*, *C. pepo*, *C. moschata*, and *L. siceraria* were assessed using a Principal Components Analysis (PCA). A second PCA was performed to compare similarities in seed size between seeds of different varieties of *C. pepo* from Paris and Nerson^39^ and archaeological seeds from El Gigante. A Ward’s agglomerative hierarchical classification using Euclidean distance was used to assess the morphological relationship of *C. pepo* seeds from El Gigante to other seeds recovered from paleontological and archaeological sites from North America (Supplementary Table S3). The cluster analysis was conducted using maximum length, maximum width, and length/width ratio. A Linear Discriminant Analysis (LDA) was performed to explore differences in seed shape between modern seeds of *C. pepo*, *C. pepo*, *C. moschata*, and *L. siceraria*. LDA is a statistical classification method used to evaluate shape variation of seeds by maximizing differences between predefined groups (i.e., species, subspecies and varieties) compared to intragroup variation.

### Supplementary Information

Supplementary Information.

## Data Availability

The datasets generated during the current study are provided in the Supplementary Information. The archeological remains studied in this research are curated in the Department of Anthropology (Pennsylvania State University).

## References

[1] Schaffer AA, Paris HS, Caballero B (2003). Melons, squashes, and gourds. Encyclopedia of Food Sciences and Nutrition.

[2] Chomicki G, Schaefer H, Renner SS (2020). Origin and domestication of Cucurbitaceae crops: Insights from phylogenies, genomics and archaeology. N. Phytol..

[3] Erickson DL, Smith BD, Clarke AC, Sandweiss DH, Tuross N (2005). An Asian origin for a 10,000-year-old domesticated plant in the Americas. Proc. Natl. Acad. Sci..

[4] Kistler L (2015). Gourds and squashes (*Cucurbita* spp.) adapted to megafaunal extinction and ecological anachronism through domestication. Proc. Natl. Acad. Sci..

[5] Decker DS, Newsom LA (1988). Numerical analysis of archaeological *Cucurbita pepo* seeds from Hontoon Island, Florida. J. Ethnobiol..

[6] Piperno DR (2009). Identifying crop plants with phytoliths (and starch grains) in Central and South America: A review and an update of the evidence. Quat. Int..

[7] Dillehay TD, Rossen J, Andres TC, Williams DE (2007). Preceramic adoption of peanut, squash, and cotton in northern Peru. Science.

[8] Kistler L (2014). Transoceanic drift and the domestication of African bottle gourds in the Americas. Proc. Natl. Acad. Sci..

[9] Paris HS (2001). History of the cultivar-groups of *Cucurbita pepo*. Horticult. Rev..

[10] Sanjur OI, Piperno DR, Andres TC, Wessel-Beaver L (2002). Phylogenetic relationships among domesticated and wild species of *Cucurbita* (Cucurbitaceae) inferred from a mitochondrial gene: Implications for crop plant evolution and areas of origin. PNAS.

[11] Smith BD (1997). Reconsidering the Ocampo caves and the era of incipient cultivation in Mesoamerica. Latin Am. Antiq..

[12] Smith BD (1997). The initial domestication of *Cucurbita pepo* in the Americas 10,000 years ago. Science.

[13] Smith BD, Smith BD (2006). Seed size increase as a marker of domestication in squash. Documenting Domestication.

[14] Smith BD (2006). Eastern North America as an independent center of plant domestication. Proc. Natl. Acad. Sci..

[15] Smith BD (2005). Reassessing Coxcatlan cave and the early history of domesticated plants in Mesoamerica. Proc. Natl. Acad. Sci..

[16] Kates HR, Soltis PS, Soltis DE (2017). Evolutionary and domestication history of *Cucurbita* (pumpkin and squash) species inferred from 44 nuclear loci. Mol. Phylogenet. Evol..

[17] Piperno DR, Ranere AJ, Holst I, Iriarte J, Dickau R (2009). Starch grain and phytolith evidence for early ninth millennium B.P. maize from the Central Balsas River Valley, Mexico. Proc. Natl. Acad. Sci..

[18] Fritz GJ (1994). Precolumbian *Cucurbita argyrosperma* spp. *argyrosperma* (Cucurbitaceae) in the eastern woodlands of North America. Econ. Bot..

[19] Fritz GJ (1986). Prehistoric Ozark Agriculture: The University of Arkansas Rockshelter Collections.

[20] Lombardo U (2020). Early Holocene crop cultivation and landscape modification in Amazonia. Nature.

[21] Piperno DR, Dillehay TD (2008). Starch grains on human teeth reveal early broad crop diet in northern Peru. PNAS.

[22] Piperno DR (2011). The origins of plant cultivation and domestication in the new world tropics: Patterns, process, and new developments. Curr. Anthropol..

[23] Ucchesu M (2016). Predictive method for correct identification of archaeological charred grape seeds: Support for advances in knowledge of grape domestication process. PLoS ONE.

[24] Scheffler TE, Hirth KG, Hasemann G (2012). The El Gigante rockshelter: Preliminary observations on an early to late Holocene occupation in southern Honduras. Latin Am. Antiq..

[25] Scheffler TE (2008). The El Gigante Rock Shelter, Honduras.

[26] Kennett DJ (2023). Trans-Holocene Bayesian chronology for tree and field crop use from El Gigante Rockshelter, Honduras. PLoS ONE.

[27] Iceland HB, Hirth KG, Lohse JC, Borejsza A, Joyce AA (2021). The Paleoindian to archaic transition in central America: Esperanza phase projectile points recovered at the El Gigante rockshelter site, Honduras. Preceramic Mesoamerica.

[28] Blake LW, Blake LW, Cutler HC (2001). Cultivated plant remains from historic Indian and Osage Indian sites. Plants from the Past.

[29] Chmurny WW (1973). The Ecology of the Middle-Mississippian Occupation of the American Bottom.

[30] Cowan CW, Gremillion KJ (1997). Evolutionary changes associated with the domestication of *Cucurbita pepo*: Evidence from eastern Kentucky. People, Places, and Landscapes: Studies in Paleoethnobotany.

[31] Lovis WA, Monaghan GW, Lovis WA, Monaghan GW (2008). Chronology and evolution of the green point flood plain and associated *Cucurbita pepo*. Current Northeast Paleoethnobotany II.

[32] Simon ML (2011). Evidence for variability among squash seeds from the Hoxie site (11CK4), Illinois. J. Archaeol. Sci..

[33] Perkl BE (1998). *Cucurbita pepo* from King Coulee, Southeastern Minnesota. Am. Antiq..

[34] King FB, Ford RI (1985). Early cultivated cucurbits in eastern North America. Prehistoric Food Production in North America.

[35] Kay M, King FB, Robinson CK (1980). Cucurbits from Phillips Spring: New evidence and interpretations. Am. Antiq..

[36] Blake LW, Cutler C, Blake LW, Cutler C (1974). Plant remains from the Rhoads site (11LO8), Illinois. Plants from the Past.

[37] Ozker D (1982). An Early Woodland Community at the Schultz Site 20SA2 in the Saginaw Valley and the Nature of the Early Woodland Adaptation in the Great Lakes Region.

[38] Blake LW, Cutler H (1982). Plant remains from the King Hill Site (23BN1) and comparisons with those from the Utz site (23SA2). Missouri Archaeol..

[39] Paris HS, Nerson H (2003). Seed dimensions in the subspecies and cultivar-groups of *Cucurbita pepo*. Genet. Resour. Crop Evol..

[40] Pope NS (2023). The expansion of agriculture has shaped the recent evolutionary history of a specialized squash pollinator. Proc. Natl. Acad. Sci..

[41] Martínez A (2018). Multidisciplinary studies in *Cucurbita maxima* (squash) domestication. Veg. Hist. Archaeobot..

[42] Lema VS (2015). Non-domesticated cultivation in the Andes: Plant management and nurturing in the Argentine northwest. Veg. Hist. Archaeobot..

[43] Duncan NA, Pearsall DM, Benfer RA (2009). Gourd and squash artifacts yield starch grains of feasting foods from preceramic Peru. Proc. Natl. Acad. Sci..

[44] Smith BD, Feinman GM, Mansanilla L (2000). Guilá Naquitz revisited: Agricultural origins in Oaxaca, Mexico. Cultural Evolution: Contemporary Viewpoints.

[45] Spengler RN, Mueller NG (2019). Grazing animals drove domestication of grain crops. Nat. Plants.

[46] Terral J-F (2004). Historical biogeography of olive domestication (*Olea europaea* L.) as revealed by geometrical morphometry applied to biological and archaeological material. J. Biogeogr..

[47] Terral J-F (2012). Insights into the historical biogeography of the date palm (*Phoenix dactylifera* L.) using geometric morphometry of modern and ancient seeds. J. Biogeogr..

[48] Orrù M, Grillo O, Lovicu G, Venora G, Bacchetta G (2013). Morphological characterization of *Vitis vinifera* L. seeds by image analysis and comparison with archaeological remains. Veg. Hist. Archaeobot..

[49] Ros J, Evin A, Bouby L, Ruas M-P (2014). Geometric morphometric analysis of grain shape and the identification of two-rowed barley (*Hordeum vulgare* subsp. *distichum* L.) in southern France. J. Archaeol. Sci..

[50] Bonhomme V (2021). Seed morphology uncovers 1500 years of vine agrobiodiversity before the advent of the Champagne wine. Sci. Rep..

[51] Thompson K, Band SR, Hodgson JG (1993). Seed size and shape predict persistence in soil. Funct. Ecol..

[52] Schwienbacher E, Marcante S, Erschbamer B (2010). Alpine species seed longevity in the soil in relation to seed size and shape—A 5-year burial experiment in the Central Alps. Flora Morphol. Distrib. Funct. Ecol. Plants.

[53] Zhao L-P, Wu G-L, Cheng J-M (2011). Seed mass and shape are related to persistence in a sandy soil in northern China. Seed Sci. Res..

[54] Spencer LJ, Snow AA (2001). Fecundity of transgenic wild-crop hybrids of *Cucurbita pepo* (Cucurbitaceae): Implications for crop-to-wild gene flow. Heredity.

[55] Rue DJ (1989). Archaic Middle American agriculture and settlement: Recent pollen data from Honduras. J. Field Archaeol..

[56] Rue D, Webster D, Traverse A (2002). Late Holocene fire and agriculture in the Copan Valley, Honduras. Ancient Mesoam..

[57] Kennett DJ (2017). High-precision chronology for Central American maize diversification from El Gigante rockshelter, Honduras. Proc. Natl. Acad. Sci..

[58] Kistler L (2020). Archaeological Central American maize genomes suggest ancient gene flow from South America. Proc. Natl. Acad. Sci..

[59] Lim TK, Lim TK, Lim TK (2012). *Cucurbita moschata*. Edible Medicinal and Non-medicinal Plants: Volume 2, Fruits.

[60] Vestal PA (1938). *Cucurbita moschata* found in pre-Columbian mounds in Guatemala. Bot. Mus. Leafl. Harvard Univ..

[61] Abràmoff MD, Magalhães PJ, Ram SJ (2004). Image processing with ImageJ. Biophoton. Int..

[62] Iwata H, Ukai Y (2002). SHAPE: A computer program package for quantitative evaluation of biological shapes based on elliptic Fourier descriptors. J. Hered..

