# Supplementary material for: Archaeobotanical evidence supports indigenous cucurbit long-term use in the Mesoamerican Neotropics
Source: Sci Rep. 2024 May 13;14:10885. doi: 10.1038/s41598-024-60723-1 (PMC11091142; doi:10.1038/s41598-024-60723-1)
Supplement: Supplementary file 1 — Supplementary Information. [file 41598_2024_60723_MOESM1_ESM.docx]

**Archaeobotanical Evidence Supports Cucurbit Domestication Origins in the Mesoamerican Neotropics**

Alejandra I. Domic^a,b,1^, Amber M. VanDerwarker^c^, Heather B. Thakar^d^, Kenneth Hirth^a^, José M. Capriles^a^, Thomas Harper^a^, Timothy E. Scheffler^e^, Logan Kistler^f^ & Douglas J. Kennett^c^

^a^Department of Anthropology, The Pennsylvania State University, University Park, PA 16802

^b^Department of Geosciences, The Pennsylvania State University, University Park, PA 16802

^c^Department of Anthropology, University of California, Santa Barbara, CA 93106

^d^Department of Anthropology, Texas A&M University, College Station, TX 77843

^e^tesARCH Services, Volcano, HI 96785

^f^Department of Anthropology, Smithsonian Institution, DC 20560

^1^Corresponding author: aid4@psu.edu

**Scientific Reports** <https://doi.org/10.1038/s41598-024-60723-1>

**Supporting Information**

Fig. S1 Cross-sections of (**a)** *Cucurbita* and (**b)** *Lagenaria* rinds recovered from El Gigante Rockshelter, Honduras.


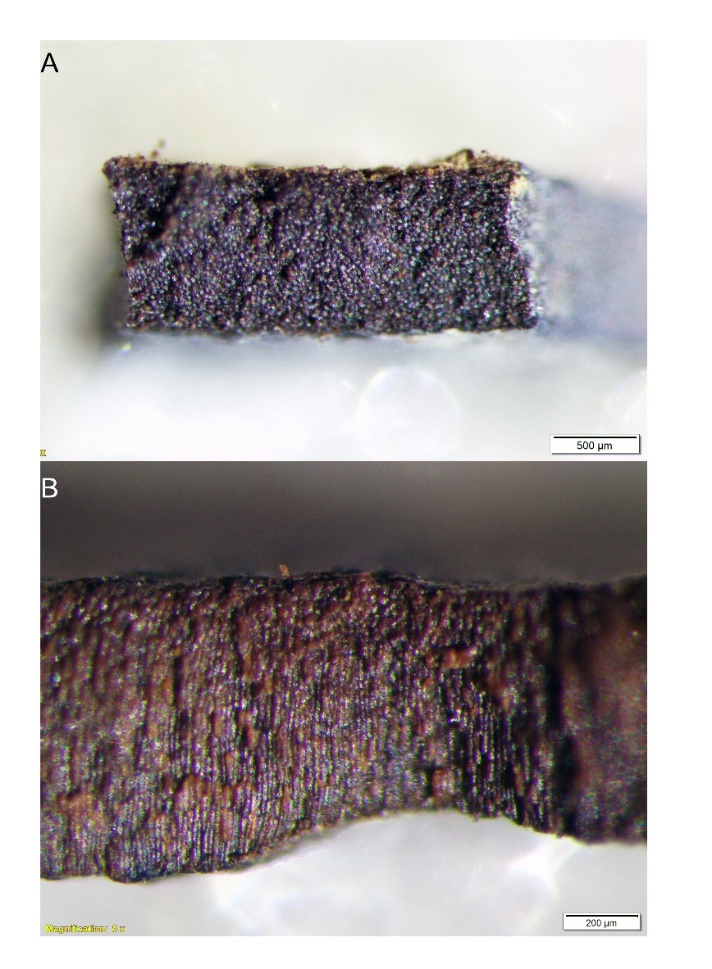


Table S1. Seed measurements of modern domesticated and wild Cucurbit species.

| **Species** | **Accession No** | **Area (mm^2^)** | **Maximum length (mm)** | **Maximum width (mm)** | **Margin width (mm)** | **Mass (g)** | **Length to width ratio (mm)** |
| --- | --- | --- | --- | --- | --- | --- | --- |
| Cucurbita pepo subsp. texana | PI 614688 | 46.28 | 9.68 | 6.57 | 0.75 | 0.40 | 1.47 |
| Cucurbita pepo subsp. texana | PI 614688 | 44.26 | 9.35 | 6.51 | 0.89 | 0.04 | 1.44 |
| Cucurbita pepo subsp. texana | PI 614688 | 33.26 | 8.19 | 5.54 | 0.64 | 0.03 | 1.48 |
| Cucurbita pepo subsp. texana | PI 614688 | 46.50 | 9.79 | 6.36 | 0.91 | 0.04 | 1.54 |
| Cucurbita pepo subsp. texana | PI 614688 | 44.79 | 9.89 | 6.17 | 0.98 | 0.04 | 1.60 |
| Cucurbita pepo subsp. texana | PI 614688 | 39.07 | 9.24 | 5.82 | 0.81 | 0.02 | 1.59 |
| Cucurbita pepo subsp. texana | PI 614688 | 34.33 | 8.55 | 5.59 | 0.83 | 0.03 | 1.53 |
| Cucurbita pepo subsp. texana | PI 614688 | 41.90 | 9.39 | 6.16 | 0.77 | 0.03 | 1.53 |
| Cucurbita pepo subsp. texana | PI 614688 | 38.42 | 9.27 | 5.68 | 0.66 | 0.03 | 1.63 |
| Cucurbita pepo subsp. texana | PI 614688 | 41.97 | 9.33 | 6.22 | 0.89 | 0.03 | 1.50 |
| Cucurbita pepo subsp. texana | PI 614688 | 45.61 | 9.29 | 6.71 | 0.95 | 0.04 | 1.38 |
| Cucurbita pepo subsp. texana | PI 614688 | 42.47 | 9.41 | 6.17 | 0.90 | 0.04 | 1.52 |
| Cucurbita pepo subsp. texana | PI 614688 | 46.57 | 9.81 | 6.43 | 0.92 | 0.04 | 1.53 |
| Cucurbita pepo subsp. texana | PI 614688 | 42.21 | 9.52 | 6.13 | 0.76 | 0.04 | 1.55 |
| Cucurbita pepo subsp. texana | PI 614688 | 23.76 | 8.79 | 5.81 | 0.79 | 0.01 | 1.51 |
| Cucurbita pepo subsp. texana | PI 614688 | 34.68 | 8.32 | 5.57 | 0.98 | 0.02 | 1.49 |
| Cucurbita pepo subsp. texana | PI 614688 | 38.29 | 9.19 | 5.44 | 0.66 | 0.02 | 1.69 |
| Cucurbita pepo subsp. texana | PI 614688 | 42.88 | 9.33 | 6.29 | 0.98 | 0.04 | 1.48 |
| Cucurbita pepo subsp. texana | PI 614688 | 38.81 | 9.52 | 5.65 | 0.79 | 0.03 | 1.68 |
| Cucurbita pepo subsp. texana | PI 614688 | 37.43 | 9.12 | 5.78 | 0.62 | 0.02 | 1.58 |
| Cucurbita pepo subsp. texana | PI 614688 | 45.31 | 9.67 | 6.55 | 0.83 | 0.03 | 1.48 |
| Cucurbita pepo subsp. texana | PI 614688 | 32.69 | 7.96 | 5.53 | 0.85 | 0.02 | 1.44 |
| Cucurbita pepo subsp. texana | PI 614688 | 46.91 | 10.05 | 6.56 | 0.85 | 0.04 | 1.53 |
| Cucurbita pepo subsp. texana | PI 614688 | 39.03 | 9.49 | 5.69 | 0.77 | 0.03 | 1.67 |
| Cucurbita pepo subsp. texana | PI 614688 | 43.63 | 9.58 | 6.26 | 0.93 | 0.03 | 1.53 |
| Cucurbita spp. pepo var. ozarkana | Ames 26885 | 54.70 | 10.59 | 6.38 | 0.02 | 0.06 | 1.66 |
| Cucurbita spp. pepo var. ozarkana | Ames 26885 | 52.81 | 10.20 | 7.23 | 0.01 | 0.04 | 1.41 |
| Cucurbita spp. pepo var. ozarkana | Ames 26885 | 50.50 | 10.14 | 7.26 | 0.01 | 0.05 | 1.40 |
| Cucurbita spp. pepo var. ozarkana | Ames 26885 | 58.49 | 10.36 | 6.87 | 0.02 | 0.06 | 1.51 |
| Cucurbita spp. pepo var. ozarkana | Ames 26885 | 44.20 | 10.90 | 7.53 | 0.01 | 0.06 | 1.45 |
| Cucurbita spp. pepo var. ozarkana | Ames 26885 | 39.71 | 9.99 | 6.28 | 0.02 | 0.06 | 1.59 |
| Cucurbita spp. pepo var. ozarkana | Ames 26885 | 48.91 | 9.36 | 5.92 | 0.02 | 0.07 | 1.58 |
| Cucurbita spp. pepo var. ozarkana | Ames 26885 | 54.76 | 10.27 | 7.11 | 0.01 | 0.06 | 1.44 |
| Cucurbita spp. pepo var. ozarkana | Ames 26885 | 39.02 | 9.72 | 5.62 | 0.02 | 0.03 | 1.73 |
| Cucurbita spp. pepo var. ozarkana | Ames 26885 | 34.63 | 9.44 | 4.99 | 0.02 | 0.04 | 1.89 |
| Cucurbita spp. pepo var. ozarkana | AMES 26875 | 48.76 | 10.35 | 6.43 | 0.02 | 0.05 | 1.61 |
| Cucurbita spp. pepo var. ozarkana | AMES 26875 | 53.20 | 10.99 | 6.90 | 0.02 | 0.07 | 1.59 |
| Cucurbita spp. pepo var. ozarkana | AMES 26875 | 42.57 | 9.81 | 6.07 | 0.02 | 0.05 | 1.62 |
| Cucurbita spp. pepo var. ozarkana | AMES 26875 | 48.47 | 10.31 | 6.15 | 0.02 | 0.05 | 1.68 |
| Cucurbita spp. pepo var. ozarkana | AMES 26875 | 42.17 | 9.50 | 6.01 | 0.02 | 0.04 | 1.58 |
| Cucurbita spp. pepo var. ozarkana | AMES 26875 | 48.31 | 10.27 | 6.52 | 0.02 | 0.05 | 1.58 |
| Cucurbita spp. pepo var. ozarkana | AMES 26875 | 49.78 | 9.36 | 7.31 | 0.01 | 0.04 | 1.28 |
| Cucurbita spp. pepo var. ozarkana | AMES 26875 | 46.00 | 9.62 | 6.57 | 0.01 | 0.05 | 1.46 |
| Cucurbita spp. pepo var. ozarkana | AMES 26875 | 51.83 | 10.23 | 6.77 | 0.02 | 0.02 | 1.51 |
| Cucurbita spp. pepo var. ozarkana | AMES 26875 | 47.66 | 10.25 | 6.49 | 0.02 | 0.05 | 1.58 |
| Cucurbita spp. pepo var. ozarkana | AMES 26877 | 38.41 | 8.71 | 6.09 | 0.01 | 0.04 | 1.43 |
| Cucurbita spp. pepo var. ozarkana | AMES 26877 | 48.79 | 9.98 | 6.82 | 0.01 | 0.05 | 1.46 |
| Cucurbita spp. pepo var. ozarkana | AMES 26877 | 57.19 | 10.64 | 7.11 | 0.01 | 0.05 | 1.50 |
| Cucurbita spp. pepo var. ozarkana | AMES 26877 | 51.52 | 10.29 | 6.94 | 0.01 | 0.06 | 1.48 |
| Cucurbita spp. pepo var. ozarkana | AMES 26877 | 40.89 | 9.54 | 6.03 | 0.02 | 0.05 | 1.58 |
| Cucurbita pepo var. fraterna | PI 614683 | 38.65 | 9.05 | 6.04 | 0.87 | 0.04 | 1.50 |
| Cucurbita pepo var. fraterna | PI 614683 | 66.03 | 12.47 | 7.43 | 1.23 | 0.08 | 1.68 |
| Cucurbita pepo var. fraterna | PI 614683 | 60.70 | 11.24 | 7.38 | 1.00 | 0.08 | 1.52 |
| Cucurbita pepo var. fraterna | PI 614683 | 61.61 | 12.12 | 7.14 | 1.12 | 0.08 | 1.70 |
| Cucurbita pepo var. fraterna | PI 614683 | 70.51 | 12.90 | 7.72 | 1.03 | 0.05 | 1.67 |
| Cucurbita pepo var. fraterna | PI 614683 | 49.59 | 10.36 | 6.59 | 0.91 | 0.06 | 1.57 |
| Cucurbita pepo var. fraterna | PI 614683 | 68.40 | 12.52 | 7.93 | 1.21 | 0.09 | 1.58 |
| Cucurbita pepo var. fraterna | PI 614683 | 38.86 | 8.97 | 5.79 | 0.87 | 0.04 | 1.55 |
| Cucurbita pepo var. fraterna | PI 614683 | 30.43 | 7.95 | 5.08 | 0.70 | 0.03 | 1.56 |
| Cucurbita pepo var. fraterna | PI 614683 | 60.96 | 12.02 | 7.22 | 0.91 | 0.07 | 1.67 |
| Cucurbita pepo var. fraterna | PI 614683 | 58.96 | 11.34 | 7.24 | 0.90 | 0.07 | 1.57 |
| Cucurbita pepo var. fraterna | PI 614683 | 74.02 | 13.12 | 7.70 | 0.96 | 0.09 | 1.70 |
| Cucurbita pepo var. fraterna | PI 614683 | 61.91 | 12.14 | 7.15 | 0.94 | 0.08 | 1.70 |
| Cucurbita pepo var. fraterna | PI 614683 | 65.16 | 12.03 | 7.14 | 1.06 | 0.08 | 1.69 |
| Cucurbita pepo var. fraterna | PI 614683 | 57.20 | 11.43 | 6.97 | 1.00 | 0.03 | 1.64 |
| Cucurbita pepo var. fraterna | PI 614683 | 28.68 | 8.23 | 4.80 | 0.79 | 0.03 | 1.71 |
| Cucurbita pepo var. fraterna | PI 614683 | 59.08 | 11.70 | 7.20 | 1.22 | 0.07 | 1.62 |
| Cucurbita pepo var. fraterna | PI 614683 | 43.59 | 12.32 | 7.67 | 1.29 | 0.10 | 1.61 |
| Cucurbita pepo var. fraterna | PI 614683 | 41.08 | 9.06 | 6.28 | 0.98 | 0.09 | 1.44 |
| Cucurbita pepo var. fraterna | PI 614683 | 52.27 | 11.17 | 6.66 | 1.11 | 0.06 | 1.68 |
| Cucurbita pepo var. fraterna | PI 614683 | 61.96 | 12.24 | 7.05 | 1.02 | 0.08 | 1.74 |
| Cucurbita pepo var. fraterna | PI 614683 | 98.79 | 14.74 | 9.38 | 1.20 | 0.10 | 1.57 |
| Cucurbita pepo var. fraterna | PI 614683 | 32.31 | 8.77 | 5.11 | 0.84 | 0.20 | 1.72 |
| Cucurbita pepo var. fraterna | PI 614683 | 62.89 | 12.13 | 7.27 | 1.00 | 0.10 | 1.67 |
| Cucurbita pepo var. fraterna | PI 614683 | 58.00 | 11.67 | 6.91 | 1.07 | 0.80 | 1.69 |
| Cucurbita pepo var. fraterna | PI 532356 | 34.88 | 8.83 | 5.23 | 0.02 | 0.03 | 1.69 |
| Cucurbita pepo var. fraterna | PI 532356 | 30.30 | 8.70 | 4.86 | 0.02 | 0.02 | 1.79 |
| Cucurbita pepo var. fraterna | PI 532356 | 33.66 | 9.04 | 5.22 | 0.02 | 0.03 | 1.73 |
| Cucurbita pepo var. fraterna | PI 532356 | 28.58 | 8.54 | 4.44 | 0.02 | 0.01 | 1.92 |
| Cucurbita pepo var. fraterna | PI 532356 | 28.62 | 8.56 | 4.64 | 0.02 | 0.02 | 1.84 |
| Cucurbita pepo var. fraterna | PI 532356 | 29.39 | 8.38 | 4.81 | 0.02 | 0.02 | 1.74 |
| Cucurbita pepo var. fraterna | PI 532356 | 33.09 | 9.01 | 5.13 | 0.02 | 0.03 | 1.75 |
| Cucurbita pepo var. fraterna | PI 532356 | 35.18 | 8.82 | 5.44 | 0.02 | 0.02 | 1.62 |
| Cucurbita pepo var. fraterna | PI 532356 | 36.57 | 9.78 | 5.33 | 0.02 | 0.02 | 1.83 |
| Cucurbita pepo var. fraterna | PI 532356 | 29.94 | 8.43 | 4.72 | 0.02 | 0.02 | 1.78 |
| Cucurbita pepo | AMES 21644 | 133.30 | 21.70 | 8.20 | 1.93 | 0.13 | 2.65 |
| Cucurbita pepo | AMES 21644 | 115.29 | 18.89 | 8.28 | 1.59 | 0.16 | 2.28 |
| Cucurbita pepo | AMES 21644 | 122.71 | 18.97 | 8.74 | 1.58 | 0.18 | 2.17 |
| Cucurbita pepo | AMES 21644 | 114.64 | 19.26 | 7.95 | 1.31 | 0.17 | 2.42 |
| Cucurbita pepo | AMES 21644 | 108.58 | 17.79 | 8.03 | 1.33 | 0.11 | 2.22 |
| Cucurbita pepo | PI 214122 1 | 118.37 | 18.91 | 8.34 | 0.96 | 0.16 | 2.27 |
| Cucurbita pepo | PI 214122 1 | 119.48 | 18.36 | 8.84 | 0.72 | 0.18 | 2.08 |
| Cucurbita pepo | PI 214122 1 | 116.99 | 18.06 | 8.62 | 1.05 | 0.17 | 2.10 |
| Cucurbita pepo | PI 214122 1 | 103.44 | 16.28 | 8.65 | 0.96 | 0.15 | 1.88 |
| Cucurbita pepo | PI 214122 1 | 94.99 | 16.55 | 7.83 | 0.77 | 0.12 | 2.11 |
| Cucurbita pepo | PI 214122 1 | 141.73 | 20.82 | 9.13 | 0.74 | 0.24 | 2.28 |
| Cucurbita pepo | PI 214122 1 | 69.85 | 11.91 | 7.81 | 0.90 | 0.08 | 1.53 |
| Cucurbita pepo | PI 214122 1 | 68.15 | 13.37 | 8.23 | 1.03 | 0.16 | 1.63 |
| Cucurbita pepo | PI 214122 1 | 64.73 | 12.41 | 7.32 | 0.94 | 0.10 | 1.70 |
| Cucurbita pepo | PI 214122 1 | 79.77 | 12.96 | 8.36 | 0.91 | 0.11 | 1.55 |
| Cucurbita pepo | PI 214122 1 | 81.69 | 12.17 | 6.96 | 0.98 | 0.14 | 1.75 |
| Cucurbita pepo | PI 458750 | 121.01 | 17.42 | 9.35 | 1.55 | 0.21 | 1.86 |
| Cucurbita pepo | PI 458750 | 121.46 | 18.11 | 9.36 | 1.42 | 0.20 | 1.93 |
| Cucurbita pepo | PI 458750 | 134.56 | 17.70 | 10.20 | 1.55 | 0.20 | 1.73 |
| Cucurbita pepo | PI 458750 | 130.45 | 18.66 | 9.45 | 1.27 | 0.19 | 1.97 |
| Cucurbita pepo | PI 318826 | 109.62 | 16.77 | 8.99 | 1.17 | 0.20 | 1.86 |
| Cucurbita pepo | PI 318826 | 119.97 | 17.81 | 9.20 | 0.89 | 0.20 | 1.94 |
| Cucurbita pepo | PI 318826 | 123.38 | 18.18 | 8.98 | 1.28 | 0.20 | 2.02 |
| Cucurbita pepo | PI 318826 | 123.36 | 17.68 | 9.88 | 1.32 | 0.30 | 1.79 |
| Cucurbita pepo | PI 318826 | 116.18 | 16.26 | 9.68 | 1.21 | 0.21 | 1.68 |
| Cucurbita pepo | PI 274336 | 95.59 | 15.78 | 8.23 | 1.40 | 0.10 | 1.92 |
| Cucurbita pepo | PI 274336 | 95.68 | 17.26 | 8.48 | 1.21 | 0.16 | 2.03 |
| Cucurbita pepo | PI 274336 | 105.43 | 13.18 | 6.70 | 1.23 | 0.10 | 1.97 |
| Cucurbita pepo | PI 274336 | 108.34 | 16.33 | 9.18 | 1.17 | 0.17 | 1.78 |
| Cucurbita pepo | PI 274336 | 65.56 | 14.54 | 8.58 | 1.11 | 0.15 | 1.70 |
| Cucurbita pepo | PI 615122 | 142.59 | 21.85 | 9.03 | 1.15 | 0.18 | 2.42 |
| Cucurbita pepo | PI 615122 | 131.54 | 21.68 | 8.16 | 1.41 | 0.19 | 2.66 |
| Cucurbita pepo | PI 615122 | 153.43 | 21.83 | 9.42 | 1.76 | 0.21 | 2.32 |
| Cucurbita pepo | PI 615122 | 143.56 | 22.87 | 8.64 | 1.33 | 0.18 | 2.65 |
| Cucurbita pepo | PI 615122 | 142.05 | 22.71 | 8.28 | 1.45 | 0.19 | 2.74 |
| Cucurbita pepo | GN 8484 | 158.81 | 21.32 | 10.19 | 0.02 | 0.17 | 2.09 |
| Cucurbita pepo | GN 8484 | 150.70 | 21.24 | 10.18 | 0.02 | 0.17 | 2.09 |
| Cucurbita pepo | GN 8484 | 104.79 | 17.75 | 8.02 | 0.02 | 0.13 | 2.21 |
| Cucurbita pepo | GN 8484 | 127.87 | 20.04 | 8.40 | 0.02 | 0.17 | 2.38 |
| Cucurbita pepo | GN 8484 | 150.49 | 21.12 | 9.57 | 0.02 | 0.10 | 2.21 |
| Cucurbita pepo | PI 615117 | 85.73 | 13.65 | 8.51 | 0.02 | 0.14 | 1.60 |
| Cucurbita pepo | PI 615117 | 93.08 | 14.29 | 8.60 | 0.02 | 0.08 | 1.66 |
| Cucurbita pepo | PI 615117 | 81.06 | 13.80 | 8.08 | 0.02 | 0.11 | 1.71 |
| Cucurbita pepo | PI 615117 | 73.64 | 12.17 | 7.84 | 0.02 | 0.12 | 1.55 |
| Cucurbita pepo | PI 615117 | 78.25 | 13.84 | 7.75 | 0.02 | 0.13 | 1.79 |
| Cucurbita pepo | PI 615131 | 105.87 | 15.93 | 8.70 | 0.02 | 0.11 | 1.83 |
| Cucurbita pepo | PI 615131 | 104.59 | 14.99 | 9.31 | 0.02 | 0.14 | 1.61 |
| Cucurbita pepo | PI 615131 | 107.47 | 15.89 | 9.35 | 0.02 | 0.16 | 1.70 |
| Cucurbita pepo | PI 615131 | 112.17 | 15.87 | 9.45 | 0.02 | 0.16 | 1.68 |
| Cucurbita pepo | PI 615131 | 118.11 | 15.87 | 10.16 | 0.02 | 0.14 | 1.56 |
| Cucurbita pepo | PI 451851 | 89.02 | 15.06 | 8.08 | 0.02 | 0.13 | 1.87 |
| Cucurbita pepo | PI 451851 | 103.52 | 15.04 | 9.27 | 0.02 | 0.12 | 1.62 |
| Cucurbita pepo | PI 451851 | 93.23 | 14.37 | 8.92 | 0.02 | 0.15 | 1.61 |
| Cucurbita pepo | PI 451851 | 82.96 | 14.76 | 7.64 | 0.02 | 0.12 | 1.93 |
| Cucurbita pepo | PI 451851 | 88.26 | 14.07 | 8.29 | 0.02 | 0.15 | 1.70 |
| Cucurbita pepo | PI 449350 | 116.55 | 16.39 | 9.84 | 0.02 | 0.19 | 1.67 |
| Cucurbita pepo | PI 449350 | 118.10 | 17.43 | 8.91 | 0.02 | 0.20 | 1.96 |
| Cucurbita pepo | PI 449350 | 128.52 | 17.77 | 9.72 | 0.02 | 0.18 | 1.83 |
| Cucurbita pepo | PI 449350 | 101.03 | 16.84 | 8.10 | 0.02 | 0.17 | 2.08 |
| Cucurbita pepo | PI 449350 | 81.89 | 14.35 | 8.13 | 0.02 | 0.13 | 1.76 |
| Cucurbita pepo | PI 442311 | 151.07 | 20.97 | 9.66 | 0.02 | 0.20 | 2.17 |
| Cucurbita pepo | PI 442311 | 142.64 | 20.52 | 9.31 | 0.02 | 0.24 | 2.20 |
| Cucurbita pepo | PI 442311 | 146.39 | 20.70 | 9.72 | 0.02 | 0.20 | 2.13 |
| Cucurbita pepo | PI 442311 | 97.73 | 17.85 | 7.87 | 0.02 | 0.16 | 2.27 |
| Cucurbita pepo | PI 442311 | 102.43 | 17.55 | 7.76 | 0.02 | 0.15 | 2.26 |
| Cucurbita pepo | PI 311103 | 125.62 | 18.97 | 9.45 | 0.02 | 0.13 | 2.01 |
| Cucurbita pepo | PI 311103 | 130.75 | 18.75 | 10.04 | 0.02 | 0.19 | 1.87 |
| Cucurbita pepo | PI 311103 | 101.15 | 14.98 | 9.40 | 0.02 | 0.12 | 1.59 |
| Cucurbita pepo | PI 311103 | 119.81 | 17.73 | 8.97 | 0.02 | 0.14 | 1.98 |
| Cucurbita pepo | PI 311103 | 92.23 | 15.72 | 8.12 | 0.02 | 0.08 | 1.94 |
| Cucurbita pepo | AMES 21650 | 109.20 | 18.42 | 8.36 | 0.02 | 0.17 | 2.20 |
| Cucurbita pepo | AMES 21650 | 112.15 | 18.44 | 8.57 | 0.02 | 0.17 | 2.15 |
| Cucurbita pepo | AMES 21650 | 105.60 | 18.59 | 8.25 | 0.02 | 0.15 | 2.25 |
| Cucurbita pepo | AMES 21650 | 103.13 | 17.43 | 8.45 | 0.02 | 0.11 | 2.06 |
| Cucurbita pepo | AMES 21650 | 104.47 | 17.66 | 8.22 | 0.02 | 0.15 | 2.15 |
| Cucurbita pepo | AMES 21651 | 99.87 | 18.04 | 7.73 | 0.02 | 0.13 | 2.33 |
| Cucurbita pepo | AMES 21651 | 111.14 | 17.88 | 8.41 | 0.02 | 0.17 | 2.13 |
| Cucurbita pepo | AMES 21651 | 100.77 | 17.23 | 8.32 | 0.02 | 0.15 | 2.07 |
| Cucurbita pepo | AMES 21651 | 123.54 | 18.75 | 8.66 | 0.02 | 0.16 | 2.17 |
| Cucurbita pepo | AMES 21651 | 106.01 | 17.85 | 8.15 | 0.02 | 0.15 | 2.19 |
| Cucurbita pepo | PI 615122 | 142.88 | 22.27 | 9.28 | 0.02 | 0.18 | 2.40 |
| Cucurbita pepo | PI 615122 | 132.02 | 22.53 | 8.79 | 0.03 | 0.19 | 2.56 |
| Cucurbita pepo | PI 615122 | 153.05 | 22.44 | 9.15 | 0.02 | 0.21 | 2.45 |
| Cucurbita pepo | PI 615122 | 142.77 | 21.87 | 8.14 | 0.03 | 0.18 | 2.69 |
| Cucurbita pepo | PI 615122 | 143.91 | 23.11 | 8.44 | 0.03 | 0.19 | 2.74 |
| Cucurbita pepo | PI 318826 | 94.35 | 17.26 | 7.62 | 0.02 | 0.20 | 2.27 |
| Cucurbita pepo | PI 318826 | 118.87 | 16.85 | 9.77 | 0.02 | 0.20 | 1.72 |
| Cucurbita pepo | PI 318826 | 144.64 | 17.31 | 9.13 | 0.02 | 0.20 | 1.90 |
| Cucurbita pepo | PI 318826 | 130.36 | 18.03 | 9.77 | 0.02 | 0.30 | 1.85 |
| Cucurbita pepo | PI 318826 | 99.89 | 16.26 | 8.45 | 0.02 | 0.21 | 1.92 |
| Cucurbita pepo | AMES 21644 | 130.04 | 19.36 | 9.10 | 0.02 | 0.13 | 2.13 |
| Cucurbita pepo | AMES 216444 | 119.17 | 19.02 | 8.28 | 0.02 | 0.16 | 2.30 |
| Cucurbita pepo | AMES 216444 | 124.87 | 21.23 | 7.74 | 0.03 | 0.18 | 2.74 |
| Cucurbita pepo | AMES 21644 | 125.43 | 19.02 | 8.85 | 0.02 | 0.17 | 2.15 |
| Cucurbita pepo | AMES 21644 | 125.78 | 21.75 | 7.62 | 0.03 | 0.11 | 2.85 |
| Cucurbita pepo | PI 615126 | 127.66 | 19.86 | 8.54 | 0.02 | 0.23 | 2.33 |
| Cucurbita pepo | PI 615126 | 127.10 | 19.79 | 8.43 | 0.02 | 0.14 | 2.35 |
| Cucurbita pepo | PI 615126 | 132.50 | 20.29 | 8.78 | 0.02 | 0.16 | 2.31 |
| Cucurbita pepo | PI 615126 | 160.31 | 21.73 | 10.13 | 0.02 | 0.20 | 2.15 |
| Cucurbita pepo | PI 615126 | 146.30 | 20.47 | 9.30 | 0.02 | 0.19 | 2.20 |
| Cucurbita moschata | PI 1738 | 89.49 | 15.23 | 8.34 | 0.76 | 0.08 | 1.83 |
| Cucurbita moschata | PI 1738 | 91.66 | 15.35 | 8.33 | 0.70 | 0.15 | 1.84 |
| Cucurbita moschata | PI 1738 | 80.77 | 14.92 | 7.48 | 0.81 | 0.11 | 1.99 |
| Cucurbita moschata | PI 1738 | 96.04 | 15.07 | 8.78 | 0.83 | 0.08 | 1.72 |
| Cucurbita moschata | PI 1738 | 80.85 | 13.98 | 8.05 | 1.06 | 0.09 | 1.74 |
| Cucurbita moschata | PI 1738 | 103.78 | 16.31 | 8.90 | 0.91 | 0.20 | 1.83 |
| Cucurbita moschata | PI 1738 | 81.91 | 14.69 | 7.41 | 0.98 | 0.15 | 1.98 |
| Cucurbita moschata | PI 1738 | 76.93 | 14.23 | 7.52 | 0.85 | 0.13 | 1.89 |
| Cucurbita moschata | PI 1738 | 73.19 | 13.97 | 7.17 | 0.81 | 0.15 | 1.95 |
| Cucurbita moschata | PI 1738 | 92.60 | 15.42 | 8.51 | 0.99 | 0.15 | 1.81 |
| Cucurbita moschata | PI 194570 | 105.72 | 17.15 | 8.24 | 1.02 | 0.15 | 2.08 |
| Cucurbita moschata | PI 194570 | 95.82 | 15.36 | 8.00 | 0.43 | 0.14 | 1.92 |
| Cucurbita moschata | PI 194570 | 105.49 | 15.75 | 8.62 | 1.00 | 0.13 | 1.83 |
| Cucurbita moschata | PI 194570 | 86.86 | 15.52 | 7.24 | 0.64 | 0.12 | 2.14 |
| Cucurbita moschata | PI 194570 | 100.90 | 16.49 | 7.89 | 1.15 | 0.14 | 2.09 |
| Cucurbita moschata | PI 262890 | 146.34 | 20.22 | 9.39 | 1.19 | 0.16 | 2.15 |
| Cucurbita moschata | PI 262890 | 148.64 | 21.64 | 9.03 | 1.30 | 0.22 | 2.40 |
| Cucurbita moschata | PI 262890 | 133.02 | 19.85 | 9.29 | 1.02 | 0.22 | 2.14 |
| Cucurbita moschata | PI 262890 | 133.25 | 19.62 | 9.38 | 1.25 | 0.22 | 2.09 |
| Cucurbita moschata | PI 262890 | 144.19 | 18.90 | 10.38 | 1.10 | 0.21 | 1.82 |
| Cucurbita moschata | PI 262890 | 134.41 | 18.01 | 9.99 | 1.32 | 0.18 | 1.80 |
| Cucurbita moschata | PI 438724 | 71.58 | 11.56 | 8.33 | 1.27 | 0.13 | 1.39 |
| Cucurbita moschata | PI 438724 | 73.19 | 12.45 | 9.04 | 0.79 | 0.14 | 1.38 |
| Cucurbita moschata | PI 438724 | 57.54 | 11.45 | 6.75 | 0.84 | 0.10 | 1.70 |
| Cucurbita moschata | PI 438724 | 61.55 | 11.19 | 7.31 | 0.93 | 0.10 | 1.53 |
| Cucurbita moschata | PI 438724 | 54.09 | 10.67 | 6.80 | 1.23 | 0.08 | 1.57 |
| Cucurbita moschata | PI 438724 | 59.25 | 11.12 | 7.35 | 0.88 | 0.06 | 1.51 |
| Cucurbita moschata | PI 172344 | 128.97 | 18.24 | 9.65 | 0.85 | 0.23 | 1.89 |
| Cucurbita moschata | PI 172344 | 111.73 | 16.97 | 9.22 | 0.98 | 0.18 | 1.84 |
| Cucurbita moschata | PI 172344 | 107.49 | 16.14 | 9.04 | 0.67 | 0.19 | 1.78 |
| Cucurbita moschata | PI 172344 | 104.47 | 16.75 | 8.73 | 1.03 | 0.20 | 1.92 |
| Cucurbita moschata | PI 172344 | 77.77 | 14.40 | 7.54 | 0.73 | 0.13 | 1.91 |
| Cucurbita moschata | PI 172344 | 81.94 | 14.63 | 7.42 | 0.74 | 0.13 | 1.97 |
| Cucurbita moschata | PI 172344 | 132.27 | 17.80 | 10.18 | 1.04 | 0.26 | 1.75 |
| Cucurbita moschata | PI 172344 | 78.20 | 15.89 | 8.75 | 0.98 | 0.14 | 1.82 |
| Cucurbita moschata | PI 172344 | 130.49 | 18.15 | 9.96 | 0.79 | 0.20 | 1.82 |
| Cucurbita moschata | PI 172344 | 76.02 | 14.26 | 7.45 | 0.66 | 0.13 | 1.91 |
| Cucurbita moschata | PI 195312 | 62.37 | 12.36 | 6.81 | 0.66 | 0.09 | 1.81 |
| Cucurbita moschata | PI 195312 | 62.53 | 12.48 | 6.87 | 0.70 | 0.09 | 1.82 |
| Cucurbita moschata | PI 195312 | 70.58 | 12.64 | 7.75 | 0.60 | 0.08 | 1.63 |
| Cucurbita moschata | PI 195312 | 60.03 | 12.09 | 6.87 | 0.56 | 0.09 | 1.76 |
| Cucurbita moschata | PI 195312 | 57.58 | 13.02 | 6.27 | 0.81 | 0.06 | 2.08 |
| Cucurbita moschata | PI 438810 | 89.99 | 14.35 | 8.49 | 0.89 | 0.09 | 1.69 |
| Cucurbita moschata | PI 438810 | 115.94 | 17.96 | 8.58 | 1.76 | 0.08 | 2.09 |
| Cucurbita moschata | PI 438810 | 42.98 | 13.26 | 8.14 | 0.82 | 0.04 | 1.63 |
| Cucurbita moschata | PI 438810 | 83.96 | 13.58 | 8.34 | 0.93 | 0.08 | 1.63 |
| Cucurbita moschata | PI 438810 | 60.32 | 11.88 | 6.97 | 0.91 | 0.03 | 1.70 |
| Cucurbita moschata | PI 438810 | 80.15 | 13.86 | 8.61 | 0.93 | 0.05 | 1.61 |
| Cucurbita moschata | PI 634700 | 132.60 | 17.52 | 10.20 | 0.02 | 0.25 | 1.34 |
| Cucurbita moschata | PI 634700 | 136.80 | 19.11 | 9.84 | 0.02 | 0.26 | 1.53 |
| Cucurbita moschata | PI 634700 | 131.64 | 18.64 | 9.51 | 0.02 | 0.27 | 1.30 |
| Cucurbita moschata | PI 634700 | 128.19 | 18.75 | 9.44 | 0.02 | 0.24 | 1.25 |
| Cucurbita moschata | PI 634700 | 132.03 | 17.08 | 10.03 | 0.02 | 0.25 | 1.40 |
| Lagenaria siceraria | PI 419089 | 64.98 | 12.15 | 6.27 | --- | 0.12 | 1.94 |
| Lagenaria siceraria | PI 419089 | 76.36 | 13.53 | 6.78 | --- | 0.14 | 2.00 |
| Lagenaria siceraria | PI 419089 | 85.86 | 15.09 | 6.90 | --- | 0.16 | 2.19 |
| Lagenaria siceraria | PI 419089 | 76.39 | 13.38 | 6.66 | --- | 0.13 | 2.01 |
| Lagenaria siceraria | PI 419089 | 68.00 | 12.96 | 6.27 | --- | 0.11 | 2.07 |
| Lagenaria siceraria | PI 702943 | 183.90 | 21.76 | 10.24 | --- | 0.18 | 2.13 |
| Lagenaria siceraria | PI 702943 | 175.31 | 22.77 | 9.66 | --- | 0.13 | 2.36 |
| Lagenaria siceraria | PI 702943 | 208.20 | 22.66 | 11.02 | --- | 0.21 | 2.06 |
| Lagenaria siceraria | PI 702943 | 213.72 | 19.17 | 12.51 | --- | 0.12 | 1.53 |
| Lagenaria siceraria | PI 358045 | 97.76 | 17.23 | 6.45 | --- | 0.17 | 2.67 |
| Lagenaria siceraria | PI 358045 | 117.46 | 19.24 | 7.50 | --- | 0.22 | 2.56 |
| Lagenaria siceraria | PI 358045 | 108.91 | 17.58 | 7.11 | --- | 0.18 | 2.47 |
| Lagenaria siceraria | PI 358045 | 91.67 | 17.71 | 6.21 | --- | 0.17 | 2.85 |
| Lagenaria siceraria | PI 358045 | 98.86 | 16.77 | 7.30 | --- | 0.21 | 2.30 |
| Lagenaria siceraria | PI 642045 | 119.70 | 19.62 | 6.80 | --- | 0.2 | 2.89 |
| Lagenaria siceraria | PI 642045 | 100.64 | 18.62 | 6.58 | --- | 0.16 | 2.83 |
| Lagenaria siceraria | PI 642045 | 132.16 | 19.76 | 7.87 | --- | 0.22 | 2.51 |
| Lagenaria siceraria | PI 642045 | 122.58 | 18.14 | 7.99 | --- | 0.24 | 2.27 |
| Lagenaria siceraria | PI 271356 | 116.28 | 17.07 | 7.74 | --- | 0.12 | 2.20 |
| Lagenaria siceraria | PI 271356 | 124.63 | 19.61 | 7.93 | --- | 0.16 | 2.47 |
| Lagenaria siceraria | PI 271356 | 120.26 | 17.35 | 8.10 | --- | 0.11 | 2.14 |
| Lagenaria siceraria | PI 271356 | 123.56 | 18.08 | 8.10 | --- | 0.14 | 2.23 |
| Lagenaria siceraria | PI 271356 | 121.19 | 19.02 | 7.57 | --- | 0.15 | 2.51 |
| Lagenaria siceraria | PI 381828 | 101.47 | 14.76 | 8.17 | --- | 0.19 | 1.81 |
| Lagenaria siceraria | PI 381828 | 107.67 | 17.02 | 7.91 | --- | 0.2 | 2.15 |
| Lagenaria siceraria | PI 381828 | 101.48 | 15.87 | 7.29 | --- | 0.19 | 2.18 |
| Lagenaria siceraria | PI 381828 | 95.56 | 15.30 | 7.20 | --- | 0.15 | 2.13 |
| Lagenaria siceraria | PI 381828 | 99.98 | 15.63 | 7.32 | --- | 0.17 | 2.13 |
| Lagenaria siceraria | PI 660978 | 66.78 | 11.61 | 7.17 | --- | 0.11 | 1.62 |
| Lagenaria siceraria | PI 660978 | 79.00 | 12.82 | 7.60 | --- | 0.15 | 1.69 |
| Lagenaria siceraria | PI 660978 | 64.02 | 10.95 | 7.14 | --- | 0.12 | 1.53 |
| Lagenaria siceraria | PI 660978 | 73.56 | 12.01 | 7.66 | --- | 0.14 | 1.57 |
| Lagenaria siceraria | PI 660978 | 72.67 | 11.88 | 7.95 | --- | 0.13 | 1.49 |

Table S2. Measurements of archeological Cucurbit seeds recovered from El Gigante Rockshelter, Honduras.

| **Species** | **Origin** | **Area (mm^2^)** | **Maximum length (mm)** | **Maximum width (mm)** | **Margin width (mm)** | **Length to width ratio (mm)** | **Mass (g)** | **Reference** |
| --- | --- | --- | --- | --- | --- | --- | --- | --- |
| Cucurbita pepo | Unit 1, Level 5 | 89.70 | 14.72 | 8.68 | 1.17 | 1.70 | 0.05 | This study |
| Cucurbita pepo | Unit 1, Level 6 | 66.15 | 12.09 | 7.26 | 0.83 | 1.67 | 0.01 | This study |
| Cucurbita pepo | Unit 1, Level 6 | 58.30 | 12.19 | 6.42 | 1.04 | 1.90 | 0.02 | This study |
| Cucurbita pepo | Unit 10, Level 1 | 97.98 | 13.47 | 10.08 | 1.78 | 1.34 | 0.02 | This study |
| Cucurbita pepo | Unit 10, Level 3 | 100.67 | 13.98 | 9.80 | 1.39 | 1.43 | 0.02 | This study |
| Cucurbita pepo | Unit 10, Level 5 | 123.49 | 16.55 | 10.65 | 1.45 | 1.55 | 0.04 | This study |
| Cucurbita pepo | Unit 10, Level 7 | 96.04 | 15.10 | 8.91 | 0.77 | 1.69 | 0.04 | This study |
| Cucurbita pepo | Unit 15, Level 1 | 84.28 | 13.65 | 8.68 | 1.27 | 1.57 | 0.01 | This study |
| Cucurbita pepo | Unit 16, Level 2 | 125.99 | 15.34 | 11.51 | 1.70 | 1.33 | 0.05 | This study |
| Cucurbita pepo | Unit 16, Level 8 | 94.22 | 15.13 | 8.99 | 1.53 | 1.68 | 0.01 | This study |
| Cucurbita pepo | Unit 17, Level 5 | 103.98 | 14.84 | 9.24 | 1.25 | 1.61 | 0.04 | This study |
| Cucurbita pepo | Unit 17, Level 6 | 86.16 | 14.25 | 8.43 | 1.22 | 1.69 | 0.04 | This study |
| Cucurbita pepo | Unit 17, Level 6 | 72.69 | 13.12 | 7.45 | 1.08 | 1.76 | 0.03 | This study |
| Cucurbita pepo | Unit 18, Level 8 | 84.26 | 14.28 | 8.38 | 1.27 | 1.70 | 0.01 | This study |
| Cucurbita pepo | Unit 2, Level 3a | 67.84 | 13.46 | 7.21 | 0.91 | 1.87 | 0.01 | This study |
| Cucurbita pepo | Unit 2, Level 4b | 58.53 | 11.66 | 6.81 | 0.82 | 1.71 | 0.06 | This study |
| Cucurbita pepo | Unit 3, Level 8 | 93.74 | 14.91 | 8.77 | 1.23 | 1.70 | 0.03 | This study |
| Cucurbita pepo | Unit 4, Level 4 | 111.77 | 14.67 | 10.36 | 1.44 | 1.42 | 0.08 | This study |
| Cucurbita pepo | Unit 4, Level 5 | 122.68 | 15.66 | 10.54 | 1.83 | 1.49 | 0.05 | This study |
| Cucurbita pepo | Unit 4, Level 5 | 49.32 | 14.30 | 9.54 | 1.28 | 1.50 | 0.05 | This study |
| Cucurbita pepo | Unit 4, Level 7 | 95.19 | 15.07 | 8.30 | 1.06 | 1.81 | 0.01 | This study |
| Cucurbita pepo | Unit 4, Level 7 | 99.71 | 15.59 | 8.59 | 1.19 | 1.81 | 0.03 | This study |
| Cucurbita pepo | Unit 4, Level 8 | 87.52 | 13.24 | 8.88 | 1.45 | 1.49 | 0.06 | This study |
| Cucurbita pepo | Unit 5, Level 3 | 100.66 | 14.71 | 8.94 | 1.34 | 1.64 | 0.02 | This study |
| Cucurbita pepo | Unit 5, Level 3 | 92.60 | 13.80 | 9.06 | 1.72 | 1.52 | 0.02 | This study |
| Cucurbita pepo | Unit 5, Level 3 | 57.57 | 10.16 | 7.44 | 0.98 | 1.37 | 0.01 | This study |
| Cucurbita pepo | Unit 5, Level 5 | 69.25 | 11.44 | 7.96 | 1.19 | 1.44 | 0.02 | This study |
| Cucurbita pepo | Unit 6, Level 13 | 95.87 | 14.76 | 8.82 | 1.24 | 1.67 | 0.05 | This study |
| Cucurbita pepo | Unit 6, Level 7a | 97.85 | 13.41 | 9.53 | 1.23 | 1.41 | 0.05 | This study |
| Cucurbita pepo | Unit 6, Level 8b | 105.66 | 15.01 | 9.11 | 1.06 | 1.65 | 0.08 | This study |
| Cucurbita pepo | Unit 7, Level 13 | 102.49 | 16.26 | 8.74 | 0.94 | 1.86 | 0.10 | This study |
| Cucurbita pepo | Unit 7, Level 2 | 75.90 | 12.40 | 8.19 | 1.32 | 1.51 | 0.04 | This study |
| Cucurbita pepo | Unit 8, Level 1 | 122.98 | 15.52 | 10.46 | 1.55 | 1.48 | 0.02 | This study |
| Cucurbita pepo | Unit 8, Level 10 | 104.60 | 13.59 | 9.92 | 1.15 | 1.37 | 0.01 | This study |
| Cucurbita pepo | Unit 8, Level 2 | 118.90 | 15.72 | 10.50 | 1.57 | 1.50 | 0.10 | This study |
| Cucurbita pepo | Unit 8, Level 2 | 110.74 | 15.50 | 10.12 | 1.74 | 1.53 | 0.02 | This study |
| Cucurbita pepo | Unit 8, Level 3 | 153.68 | 17.56 | 11.47 | 2.43 | 1.53 | 0.05 | This study |
| Cucurbita pepo | Unit 8, Level 3 | 177.57 | 19.11 | 12.66 | 1.88 | 1.51 | 0.11 | This study |
| Cucurbita pepo | Unit 8, Level 8 | 103.38 | 14.23 | 9.46 | 1.64 | 1.50 | 0.08 | This study |
| Cucurbita pepo | Unit 9, Level 4 | 112.08 | 15.42 | 10.09 | 1.43 | 1.53 | 0.04 | This study |
| Cucurbita pepo | Unit 9, Level 7 | 93.23 | 13.92 | 9.41 | 1.16 | 1.48 | 0.01 | This study |
| Cucurbita pepo | Unit 9, Level 8 | 91.16 | 14.13 | 8.75 | 0.97 | 1.62 | 0.05 | This study |
| Cucurbita pepo | Unit 3, Level 9 | 67.18 | 12.25 | 7.27 | 1.15 | 1.68 | 0.02 | This study |
| Lagenaria siceraria | Unit 6, Level 15a | 59.75 | 11.60 | 6.39 |  | 1.82 | 0.07 | This study |
| Lagenaria siceraria | Unit 12, Level 2 | 81.30 | 12.21 | 7.09 |  | 1.72 | 0.04 | This study |
| Lagenaria siceraria | Unit 13, Level 4 | 117.80 | 15.37 | 8.86 |  | 1.73 | 0.19 | This study |
| Lagenaria siceraria | Unit 3, Level 8 | 35.70 | 9.99 | 4.29 |  | 2.33 | 0.02 | This study |
| Lagenaria siceraria | Unit 3, Level 8 | 61.67 | 11.88 | 6.08 |  | 1.95 | 0.08 | This study |
| Lagenaria siceraria | Unit 6, Level 5b | 59.88 | 13.01 | 5.23 |  | 2.49 | 0.06 | This study |
| Lagenaria siceraria | Unit 12, Level 1 | 96.02 | 14.47 | 7.71 |  | 1.88 | 0.07 | This study |
| Lagenaria siceraria | Unit 9, Level 4 | 42.15 | 10.22 | 5.10 |  | 2.00 | 0.02 | This study |
| Lagenaria siceraria | Unit 17, Level 5 | 32.30 | 12.21 | 6.90 |  | 1.77 | 0.09 | This study |
| Cucurbita moschata | Unit 4, Level 5 | 138.90 | 17.10 | 11.30 | 1.20 | 1.51 | 0.05 | This study |
| Cucurbita moschata | Unit 4, Level 5 | 126.00 | 17.50 | 9.70 | 1.70 | 1.80 | 0.02 | This study |
| Cucurbita moschata | Unit 7, Level 11 | 139.65 | 16.35 | 10.50 | 1.21 | 1.56 | 0.03 | This study |
| Cucurbita moschata | Unit 4, Level 5 | 138.01 | 17.04 | 11.35 | 1.09 | 1.50 | 0.02 | This study |

Table S3. Measurements of *Cucurbita* seeds recovered from archaeological and paleontological sites from Mesoamerica and North America.

| **Species** | **Origin** | **Site** | **Maximum length (mm)** | **Maximum width (mm)** | **Length to width ratio (mm)** | **Reference** |
| --- | --- | --- | --- | --- | --- | --- |
| Cucurbita pepo | Archaeological | Brown site | 16.43 | 7.9 | 2.08 | Blacke 2001 |
| Cucurbita pepo | Archaeological | Brown site | 14.7 | 7.6 | 1.93 | Blacke (2001) |
| Cucurbita pepo | Archaeological | Brown site | 12.3 | 6.9 | 1.78 | Blacke (2001) |
| Cucurbita pepo | Archaeological | Cloudsplitter | 8.7 | 5.4 | 1.61 | Cowan, 1997 |
| Cucurbita pepo | Archaeological | Cloudsplitter | 8.7 | 5.4 | 1.61 | Cowan 1997 |
| Cucurbita pepo | Archaeological | Cloudsplitter | 12.6 | 7.4 | 1.70 | Cowan 1997 |
| Cucurbita pepo | Archaeological | Green Point | 13.9 | 8.8 | 1.58 | Wright 1964 |
| Cucurbita pepo | Archaeological | Green Point | 12.9 | 8 | 1.61 | Wright 1964 |
| Cucurbita pepo | Archaeological | Green Point | 16.4 | 8.7 | 1.89 | Lovis & Monagham 2008 |
| Cucurbita pepo | Archaeological | Guilá Naquitz | 11.4 |  |  | Smith 2006 |
| Cucurbita pepo | Archaeological | Guilá Naquitz | 12 |  |  | Smith 2006 |
| Cucurbita pepo | Archaeological | Guilá Naquitz | 12.1 |  |  | Smith 2006 |
| Cucurbita pepo | Archaeological | Guilá Naquitz | 12.5 |  |  | Smith 2006 |
| Cucurbita pepo | Archaeological | Guilá Naquitz | 13.2 |  |  | Smith 2006 |
| Cucurbita pepo | Archaeological | Guilá Naquitz | 13.8 |  |  | Smith 2006 |
| Cucurbita pepo | Archaeological | Haystack | 11.6 | 6.3 | 1.84 | Cowan 1997 |
| Cucurbita pepo | Archaeological | Hoonton Island | 9.36 | 6.14 | 1.52 | Decker & Newsom 1988 |
| Cucurbita pepo subsp. texana/ pepo subsp. ovifera | Archaeological | Hoonton Island | 9.43 | 6.36 | 1.48 | Decker & Newsom 1988 |
| Cucurbita pepo subsp. texana/ pepo subsp. ovifera | Archaeological | Hoonton Island | 11.33 | 7.76 | 1.46 | Decker & Newsom 1988 |
| Cucurbita pepo | Archaeological | Hoonton Island | 15.22 | 9.1 | 1.67 | Decker & Newsom 1988 |
| Cucurbita pepo subsp. texana | Archaeological | Hoonton Island | 15.5 | 9 | 0.58 | Decker 1986, Decker & Wilson 1986 |
| Cucurbita pepo subsp. texana | Archaeological | Hoonton Island | 9.39 | 6.05 | 0.64 | Decker 1986, Decker & Wilson 1986 |
| Cucurbita pepo subsp. texana | Archaeological | Hoonton Island | 9 | 5.85 | 0.65 | Decker 1986, Decker & Wilson 1986 |
| Cucurbita pepo subsp. texana | Archaeological | Hoonton Island | 15.22 | 9.1 | 0.60 | Decker 1986, Decker & Wilson 1986 |
| Cucurbita pepo subsp. texana | Archaeological | Hoonton Island | 9.22 | 6.13 | 0.66 | Decker 1986, Decker & Wilson 1986 |
| Cucurbita pepo subsp. texana | Archaeological | Hoonton Island | 9.39 | 6.46 | 0.69 | Decker 1986, Decker & Wilson 1986 |
| Cucurbita pepo subsp. texana | Archaeological | Hoonton Island | 8.93 | 5.9 | 0.66 | Decker 1986, Decker & Wilson 1986 |
| Cucurbita pepo subsp. texana | Archaeological | Hoonton Island | 9.07 | 6.11 | 0.67 | Decker 1986, Decker & Wilson 1986 |
| Cucurbita pepo subsp. texana | Archaeological | Hoonton Island | 10.35 | 7.1 | 0.69 | Decker 1986, Decker & Wilson 1986 |
| Cucurbita pepo subsp. texana | Archaeological | Hoonton Island | 9.28 | 6.37 | 0.69 | Decker 1986, Decker & Wilson 1986 |
| Cucurbita pepo subsp. texana | Archaeological | Hoonton Island | 11.33 | 7.76 | 0.68 | Decker 1986, Decker & Wilson 1986 |
| Cucurbita pepo subsp. texana | Archaeological | Hoonton Island | 9.43 | 6.38 | 0.68 | Decker 1986, Decker & Wilson 1986 |
| Cucurbita pepo subsp. texana | Archaeological | Hoonton Island | 9.63 | 6.47 | 0.67 | Decker 1986, Decker & Wilson 1986 |
| Cucurbita pepo subsp. texana | Archaeological | Hoonton Island | 9.54 | 6.42 | 0.67 | Decker 1986, Decker & Wilson 1986 |
| Cucurbita pepo subsp. texana | Archaeological | Hoonton Island | 11.68 | 7.64 | 0.65 | Decker 1986, Decker & Wilson 1986 |
| Cucurbita pepo subsp. texana | Archaeological | Hoonton Island | 9.55 | 6.1 | 0.64 | Decker 1986, Decker & Wilson 1986 |
| Cucurbita pepo subsp. texana | Archaeological | Hoonton Island | 9.36 | 6.14 | 0.66 | Decker 1986, Decker & Wilson 1986 |
| Cucurbita pepo subsp. texana | Archaeological | Hoonton Island | 9.14 | 6.18 | 0.68 | Decker 1986, Decker & Wilson 1986 |
| Cucurbita pepo subsp. texana | Archaeological | Hoonton Island | 9.07 | 6.3 | 0.69 | Decker 1986, Decker & Wilson 1986 |
| Cucurbita pepo subsp. texana | Archaeological | Hoonton Island | 9.01 | 6.1 | 0.68 | Decker 1986, Decker & Wilson 1986 |
| Cucurbita pepo | Archaeological | Hoonton Island | 9.94 | 6.62 | 0.67 | Decker 1986, Decker & Wilson 1986 |
| Cucurbita pepo subsp. ovifera | Archaeological | Hoxie site | 10 | 7 | 0.70 | Simon 2011 |
| Cucurbita pepo subsp. ovifera | Archaeological | Hoxie site | 10.5 | 7.5 | 0.71 | Simon 2011 |
| Cucurbita pepo subsp. ovifera | Archaeological | Hoxie site | 10.5 | 7.5 | 0.71 | Simon 2011 |
| Cucurbita pepo | Archaeological | Hoxie site | 12 | 7 | 0.58 | Simon 2011 |
| Cucurbita pepo | Archaeological | Hoxie site | 12 | 7.5 | 0.63 | Simon 2011 |
| Cucurbita pepo | Archaeological | Hoxie site | 12.3 | 7.3 | 0.59 | Simon 2011 |
| Cucurbita pepo | Archaeological | Hoxie site | 12.3 | 8.8 | 0.72 | Simon 2011 |
| Cucurbita pepo | Archaeological | Hoxie site | 12.3 | 8.8 | 0.72 | Simon 2011 |
| Cucurbita pepo | Archaeological | Hoxie site | 12.8 | 9.8 | 0.77 | Simon 2011 |
| Cucurbita pepo | Archaeological | Hoxie site | 13.8 | 8.8 | 0.64 | Simon 2011 |
| Cucurbita pepo | Archaeological | Hoxie site | 14.3 | 9.8 | 0.69 | Simon 2011 |
| Cucurbita pepo | Archaeological | Hoxie site | 14.33 | 9.8 | 0.68 | Simon 2011 |
| Cucurbita pepo | Archaeological | Hoxie site | 14.8 | 8.8 | 0.59 | Simon 2011 |
| Cucurbita pepo | Archaeological | Hoxie site | 15 | 10 | 0.67 | Simon 2011 |
| Cucurbita pepo | Archaeological | Hoxie site | 15.8 | 9.8 | 0.62 | Simon 2011 |
| Cucurbita pepo | Archaeological | Hoxie site | 18.3 | 10.8 | 0.59 | Simon 2011 |
| Cucurbita pepo | Archaeological | Hoxie site | 18.3 | 10.3 | 0.56 | Simon 2011 |
| Cucurbita pepo | Archaeological | Hoxie site | 18.3 | 11.3 | 0.62 | Simon 2011 |
| Cucurbita pepo | Archaeological | Hoxie site | 17.3 | 10.8 | 0.62 | Simon 2011 |
| Cucurbita pepo | Archaeological | Hoxie site | 16.8 | 10.8 | 0.64 | Simon 2011 |
| Cucurbita pepo | Archaeological | Hoxie site | 17 | 10 | 0.59 | Simon 2011 |
| Cucurbita pepo | Archaeological | King Coulee | 12.5 | 8.4 | 1.49 | Perkl 1998 |
| Cucurbita pepo | Archaeological | King Coulee | 12.2 | 7.6 | 1.61 | Perkl 1998 |
| Cucurbita pepo | Archaeological | King Hill | 13.6-17.3 | 7.4-9.5 |  | Blacke & Cutler 1982 |
| Cucurbita pepo | Archaeological | King Hill | 11-11.7 | 5-6.5 |  | Blacke & Cutler 1982 |
| Cucurbita | Archaeological | Mount 51, Cahokia | 9 | 6 | 1.50 | Chmurny 1973 |
| Cucurbita | Archaeological | Mount 51, Cahokia | 11 | 6 | 1.83 | Chmurny 1973 |
| Cucurbita | Archaeological | Mount 51, Cahokia | 15 | 9 | 1.67 | Chmurny 1973 |
| Cucurbita | Archaeological | Mount 51, Cahokia | 18 | 11 | 1.64 | Chmurny 1973 |
| Cucurbita pepo | Archaeological | Newt Kash Hollow | 13.1 | 9.1 | 1.44 | Cowan 1997 |
| Cucurbita pepo | Archaeological | Ozarks - Agnew Small | 10.8 | 6.3 | 1.71 | Fritz 1997 |
| Cucurbita pepo | Archaeological | Ozarks - Agnew Small | 13.2 | 7.5 | 1.76 | Fritz 1997 |
| Cucurbita pepo | Archaeological | Ozarks - Agnew Small | 12.7 | 7.8 | 1.63 | Fritz 1997 |
| Cucurbita pepo | Archaeological | Ozarks - Agnew Small | 12 | 6.6 | 1.82 | Fritz 1997 |
| Cucurbita pepo | Archaeological | Ozarks - Agnew Small | 11.8 | 8.5 | 1.39 | Fritz 1997 |
| Cucurbita pepo | Archaeological | Ozarks - Agnew Small | 11.8 | 7.8 | 1.51 | Fritz 1997 |
| Cucurbita pepo | Archaeological | Ozarks - Agnew Small | 11.3 |  |  | Fritz 1997 |
| Cucurbita pepo | Archaeological | Ozarks - Agnew Small | 10.5 | 8 | 1.31 | Fritz 1997 |
| Cucurbita pepo | Archaeological | Ozarks - Agnew Small | 9.7 | 7 | 1.39 | Fritz 1997 |
| Cucurbita pepo | Archaeological | Ozarks - Beaver Pond Large | 17.3 | 10.7 | 1.62 | Fritz 1986 |
| Cucurbita pepo | Archaeological | Ozarks - Beaver Pond Small | 13 | 8 | 1.63 | Fritz 1986 |
| Cucurbita pepo | Archaeological | Ozarks - Whitney Bluff Large | 18.5 | 9 | 2.06 | Fritz 1986 |
| Cucurbita pepo | Archaeological | Phillip Spring | 10.5 |  |  | Smith 2006 |
| Cucurbita pepo | Archaeological | Phillips Spring | 10.5 | 7.03 | 1.49 | King 1985 |
| Cucurbita pepo | Archaeological | Phillips Springs | 9.7 | 6.5 | 1.49 | Kay et al. 1980 |
| Cucurbita pepo | Archaeological | Rhoads | 16.4 | 10.3 | 1.59 | Blacke & Cutler 1974 |
| Cucurbita pepo | Archaeological | Rhoads | 10.3 | 6 | 1.72 | Blacke & Cutler 1974 |
| Cucurbita pepo | Archaeological | Rogers | 11.6 | 7.3 | 1.59 | Cowan 1997 |
| Cucurbita pepo | Archaeological | Romeros cave | 13.5 | 9.1 | 1.48 | Smith 1997a |
| Cucurbita pepo | Archaeological | Romeros cave | 12.2 | 6.67 | 1.83 | Smith 1997a |
| Cucurbita pepo | Archaeological | Romeros cave | 12.7 | 8.4 | 1.51 | Smith 1997a |
| Cucurbita pepo | Archaeological | Romeros cave | 12.5 | 8.4 | 1.49 | Smith 1997a |
| Cucurbita pepo | Archaeological | Romeros cave | 13.2 | 8.56 | 1.54 | Smith 1997a |
| Cucurbita pepo | Archaeological | Romeros cave | 12.6 | 8.2 | 1.54 | Smith 1997a |
| Cucurbita pepo | Archaeological | Romeros cave | 14.6 | 9.4 | 1.55 | Smith 1997a |
| Cucurbita pepo | Archaeological | Romeros cave | 12.7 | 8.3 | 1.53 | Smith 1997a |
| Cucurbita pepo | Archaeological | Romeros cave | 17.5 | 13 | 1.35 | Smith 1997a |
| Cucurbita pepo | Archaeological | Romeros cave | 20.5 | 13.4 | 1.53 | Smith 1997a |
| Cucurbita pepo | Archaeological | Romeros cave | 18.7 | 11.4 | 1.64 | Smith 1997a |
| Cucurbita pepo | Archaeological | Romeros cave | 18 | 10.6 | 1.70 | Smith 1997a |
| Cucurbita pepo pepo | Archaeological | Romeros cave | 18.7 | 11.7 | 1.60 | Smith 1997a |
| Cucurbita pepo subsp. ovifera | Archaeological | Saguinaw valley | 7.9 | 4 | 1.98 | Ozker 1982 |
| Cucurbita pepo | Archaeological | Salts Cave | 11.3 | 7.3 | 1.55 | King 1985 |
| Cucurbita pepo | Archaeological | Utz Site (23SA2) | 8.7 | 5 | 1.74 | Blacke & Cutler 1982 |
| Cucurbita pepo | Archaeological | Utz Site (23SA2) | 14 | 6.5 | 2.15 | Blacke & Cutler 1982 |
| Cucurbita pepo pepo | Archaeological | Valenzuelas cave | 13.9 | 9.4 | 1.48 | Smith 1997a |
| Cucurbita pepo pepo | Archaeological | Valenzuelas cave | 12.1 | 8.2 | 1.48 | Smith 1997a |
| Cucurbita pepo pepo | Archaeological | Valenzuelas cave | 15.1 | 9.2 | 1.64 | Smith 1997a |
| Cucurbita pepo pepo | Archaeological | Valenzuelas cave | 12.4 | 8.6 | 1.44 | Smith 1997a |
| Cucurbita pepo | Paleontological | Page-Ladson | 9.48 | 6.28 | 1.51 | Newsom et al. 1993 |
| Cucurbita pepo | Paleontological | Page-Ladson | 9.9 | 6.28 | 1.58 | Newsom et al. 1993 |
| Cucurbita pepo | Paleontological | Page-Ladson | 10.05 | 7.2 | 1.40 | Newsom et al. 1993 |
| Cucurbita pepo | Paleontological | Page-Ladson | 9.9 | 6.15 | 1.61 | Newsom et al. 1993 |
| Cucurbita pepo | Paleontological | Page-Ladson | 10.4 | 7.1 | 1.46 | Newsom et al. 1993 |
| Cucurbita pepo | Paleontological | Page-Ladson | 10.15 | 7.1 | 1.43 | Newsom et al. 1993 |
| Cucurbita pepo | Paleontological | Page-Ladson | 10.4 | 6.3 | 1.65 | Newsom et al. 1993 |
| Cucurbita pepo | Paleontological | Page-Ladson | 10.15 | 6.8 | 1.49 | Newsom et al. 1993 |
| Cucurbita pepo | Paleontological | Page-Ladson | 10.46 | 6.8 | 1.54 | Newsom et al. 1993 |
| Cucurbita pepo | Paleontological | Page-Ladson | 9.14 | 6.38 | 1.43 | Newsom et al. 1993 |
| Cucurbita pepo | Paleontological | Page-Ladson | 10.03 | 6.45 | 1.56 | Newsom et al. 1993 |
| Cucurbita pepo | Paleontological | Page-Ladson | 10.15 | 6.72 | 1.51 | Newsom et al. 1993 |
| Cucurbita pepo | Paleontological | Page-Ladson | 10.46 | 6.8 | 1.54 | Newsom et al. 1993 |
| Cucurbita pepo | Paleontological | Page-Ladson | 9.14 | 6.38 | 1.43 | Newsom et al. 1993 |
| Cucurbita pepo | Paleontological | Page-Ladson | 10.03 | 6.45 | 1.56 | Newsom et al. 1993 |
| Cucurbita pepo | Paleontological | Page-Ladson | 10.15 | 6.72 | 1.51 | Newsom et al. 1993 |
| Cucurbita pepo | Paleontological | Page-Ladson | 9.11 | 6.53 | 1.40 | Newsom et al. 1993 |
| Cucurbita pepo | Paleontological | Page-Ladson | 10.13 | 6.17 | 1.64 | Newsom et al. 1993 |
| Cucurbita pepo | Paleontological | Page-Ladson | 8.73 | 5.07 | 1.72 | Newsom et al. 1993 |
| Cucurbita pepo | Paleontological | Page-Ladson | 9.32 | 6.47 | 1.44 | Newsom et al. 1993 |
| Cucurbita pepo | Paleontological | Page-Ladson | 10.13 | 6.43 | 1.58 | Newsom et al. 1993 |
| Cucurbita pepo | Paleontological | Page-Ladson | 9.71 | 5.94 | 1.63 | Newsom et al. 1993 |
| Cucurbita pepo | Paleontological | Page-Ladson | 10.03 | 6.71 | 1.49 | Newsom et al. 1993 |
| Cucurbita pepo | Paleontological | Page-Ladson | 9.71 | 5.94 | 1.63 | Newsom et al. 1993 |
| Cucurbita pepo | Paleontological | Page-Ladson | 10.03 | 6.71 | 1.49 | Newsom et al. 1993 |
| Cucurbita pepo | Paleontological | Page-Ladson | 8.89 | 6.47 | 1.37 | Newsom et al. 1993 |
| Cucurbita pepo | Paleontological | Page-Ladson | 9.71 | 6.49 | 1.50 | Newsom et al. 1993 |
| Cucurbita pepo | Paleontological | Page-Ladson | 9.97 | 6.82 | 1.46 | Newsom et al. 1993 |
| Cucurbita pepo | Paleontological | Page-Ladson | 9.61 | 6.86 | 1.40 | Newsom et al. 1993 |
| Cucurbita pepo | Paleontological | Page-Ladson | 10.55 | 6.94 | 1.52 | Newsom et al. 1993 |
| Cucurbita pepo | Paleontological | Page-Ladson | 9.35 | 7 | 1.34 | Newsom et al. 1993 |
| Cucurbita pepo | Paleontological | Page-Ladson | 10.4 | 7.3 | 1.42 | Newsom et al. 1993 |
| Cucurbita pepo | Paleontological | Page-Ladson | 9.6 | 6.55 | 1.47 | Newsom et al. 1993 |
| Cucurbita pepo | Paleontological | Page-Ladson | 9.2 | 7.4 | 1.24 | Newsom et al. 1993 |
| Cucurbita pepo | Paleontological | Page-Ladson | 11.15 | 7.1 | 1.57 | Newsom et al. 1993 |
| Cucurbita pepo | Paleontological | Page-Ladson | 10.5 | 7.6 | 1.38 | Newsom et al. 1993 |
| Cucurbita pepo | Paleontological | Page-Ladson | 9.65 | 6.9 | 1.40 | Newsom et al. 1993 |

Table S4. Accession information of modern domesticated and wild Cucurbit species used in this study.

| **Species** | **Accession** | **Origin** | **Genebank** |
| --- | --- | --- | --- |
| Cucurbita moschata | PI 172344 | Mexico | S9 |
| Cucurbita moschata | PI 1738 | France |  |
| Cucurbita moschata | PI 194570 | Cochabamba, Bolivia | NC7 |
| Cucurbita moschata | PI 195312 | Guatemala | S9 |
| Cucurbita moschata | PI 262890 | Mexico | S9 |
| Cucurbita moschata | PI 438724 | Mexico | S9 |
| Cucurbita moschata | PI 438810 | Mexico | S9 |
| Cucurbita moschata | PI 634700 | Oaxaca, Mexico | S9 |
| Cucurbita pepo | AMES 21644 | Oaxaca, Mexico | NC7 |
| Cucurbita pepo | AMES 21650 | Sonora, Mexico | NC7 |
| Cucurbita pepo | AMES 21651 | Veracruz, Mexico | NC7 |
| Cucurbita pepo | GN 8484 |  |  |
| Cucurbita pepo | PI 615126 | Guerrero, Mexico | NC7 |
| Cucurbita pepo | PI 214122 | Cochabamba, Bolivia | NC7 |
| Cucurbita pepo | PI 274336 | Retalhuleu, Guatemala | NC7 |
| Cucurbita pepo | PI 311103 | Huehuetenango, Guatemala | NC7 |
| Cucurbita pepo | PI 318826 | Chihuahua, Mexico | NC7 |
| Cucurbita pepo | PI 442311 | Guanajuato, Mexico | NC7 |
| Cucurbita pepo | PI 449350 | Huehuetenango, Guatemala | NC7 |
| Cucurbita pepo | PI 451851 | Solola, Guatemala | NC7 |
| Cucurbita pepo | PI 458750 | Solola, Guatemala | NC7 |
| Cucurbita pepo | PI 615117 | Chihuahua, Mexico | NC7 |
| Cucurbita pepo | PI 615122 | Guerrero, Mexico | NC7 |
| Cucurbita pepo | PI 615131 | Chiapas, Mexico | NC7 |
| Cucurbita pepo subsp. texana | PI 614688 | Texas, United States | NC7 |
| Cucurbita pepo var. fraterna | PI 532356 | Tamaulipas, Mexico | NC7 |
| Cucurbita pepo var. fraterna | PI 614683 | Tamaulipas, Mexico | NC7 |
| Cucurbita spp. pepo var. ozarkana | AMES 26875 | Illinois, United States | NC7 |
| Cucurbita spp. pepo var. ozarkana | AMES 26877 | Illinois, United States | NC7 |
| Cucurbita spp. pepo var. ozarkana | Ames 26885 | Kentucky, United States | NC7 |
| Lagenaria siceraria | PI 702943 | California, Untied States | S9 |
| Lagenaria siceraria | PI 271356 | India | S9 |
| Lagenaria siceraria | PI 358045 | Former Serbia and Montenegro | S9 |
| Lagenaria siceraria | PI 381828 | Rajasthan, India | S9 |
| Lagenaria siceraria | PI 419089 | Beijing Shi, China | S9 |
| Lagenaria siceraria | PI 642045 | Georgia, United States | S9 |
| Lagenaria siceraria | PI 660978 | Jalisco, Mexico | S9 |
